# Time Series Transcriptomic Analysis of Bronchoalveolar Lavage Cells from Piglets Infected with Virulent or Low-Virulent Porcine Reproductive and Respiratory Syndrome Virus 1

**DOI:** 10.1128/JVI.01140-21

**Published:** 2022-02-09

**Authors:** J. M. Sánchez-Carvajal, I. M. Rodríguez-Gómez, I. Ruedas-Torres, S. Zaldívar-López, F. Larenas-Muñoz, R. Bautista-Moreno, J. J. Garrido, F. J. Pallarés, L. Carrasco, J. Gómez-Laguna

**Affiliations:** a Department of Anatomy and Comparative Pathology and Toxicology, Faculty of Veterinary Medicine, University of Córdoba, Córdoba, Spain; b Department of Genetics, Faculty of Veterinary Medicine, University of Córdoba, Córdoba, Spain; c Andalusian Platform of Bioinformatic, University of Málaga, Campanillas, Málaga, Spain; Loyola University Chicago

**Keywords:** porcine reproductive and respiratory syndrome virus, virulence, bronchoalveolar lavage cells, time-series transcriptomic analysis, Hub genes, immune checkpoints, T-cells

## Abstract

Porcine reproductive and respiratory syndrome virus (PRRSV) has evolved to escape the immune surveillance for a survival advantage leading to a strong modulation of host’s immune responses and favoring secondary bacterial infections. However, limited data are available on how the immunological and transcriptional responses elicited by virulent and low-virulent PRRSV-1 strains are comparable and how they are conserved during the infection. To explore the kinetic transcriptional signature associated with the modulation of host immune response at lung level, a time-series transcriptomic analysis was performed in bronchoalveolar lavage cells upon experimental *in vivo* infection with two PRRSV-1 strains of different virulence, virulent subtype 3 Lena strain or the low-virulent subtype 1 3249 strain. The time-series analysis revealed overlapping patterns of dysregulated genes enriched in T-cell signaling pathways among both virulent and low-virulent strains, highlighting an upregulation of co-stimulatory and co-inhibitory immune checkpoints that were disclosed as Hub genes. On the other hand, virulent Lena infection induced an early and more marked “negative regulation of immune system process” with an overexpression of co-inhibitory receptors genes related to T-cell and NK cell functions, in association with more severe lung lesion, lung viral load, and BAL cell kinetics. These results underline a complex network of molecular mechanisms governing PRRSV-1 immunopathogenesis at lung level, revealing a pivotal role of co-inhibitory and co-stimulatory immune checkpoints in the pulmonary disease, which may have an impact on T-cell activation and related pathways. These immune checkpoints, together with the regulation of cytokine-signaling pathways, modulated in a virulence-dependent fashion, orchestrate an interplay among pro- and anti-inflammatory responses.

**IMPORTANCE** Porcine reproductive and respiratory syndrome virus (PRRSV) is one of the major threats to swine health and global production, causing substantial economic losses. We explore the mechanisms involved in the modulation of host immune response at lung level performing a time-series transcriptomic analysis upon experimental infection with two PRRSV-1 strains of different virulence. A complex network of molecular mechanisms was revealed to control the immunopathogenesis of PRRSV-1 infection, highlighting an interplay among pro- and anti-inflammatory responses as a potential mechanism to restrict inflammation-induced lung injury. Moreover, a pivotal role of co-inhibitory and co-stimulatory immune checkpoints was evidenced, which may lead to progressive dysfunction of T cells, impairing viral clearance and leading to persistent infection, favoring as well secondary bacterial infections or viral rebound. However, further studies should be conducted to evaluate the functional role of immune checkpoints in advanced stages of PRRSV infection and explore a possible T-cell exhaustion state.

## INTRODUCTION

Porcine reproductive and respiratory syndrome virus (PRRSV) is the major hazard to swine health and global production, causing dramatic economic losses (1, 2) due to reproductive failure in pregnant sows and respiratory disorders in growing pigs (3, 4). PRRSV encompasses two species, *Betaarterivirus suid 1* and *Betaarterivirus suid 2* (PRRSV-1 and PRRSV-2, respectively) (5), which present a wide inter- and intra-species viral and antigenic diversity (6–8). PRRSV shows signs of a marked mutation rate, favoring the emergence and re-emergence of virulent strains worldwide, which has gained special interest since 2006. PRRSV-1 virulent strains have been reported to induce high morbidity and mortality rates, fever, hemorrhages, severe lung damage, and, eventually, lesions in other organs (9–11, 13–15), increasing the concern for understanding the immunopathology of PRRSV.

PRRSV has evolved a variety of strategies to manipulate, even to evade, the host antiviral innate immunity and some cellular survival-associated pathways, facilitating its replication and distribution (16, 17). An impairment of type I interferon (IFN-I) signaling cascade and production, modulation of cytokine expression by immune cells as well as antigen presentation and T-cell activation, has been described for PRRSV infection (16, 18–22). Indeed, this dysregulation of host’s immune responses (18, 20) is bound to persistent infection and to facilitation of secondary bacterial infections, resulting in porcine respiratory disease complex (PRDC) (23).

High-throughput RNA sequencing (RNA-seq) technology combined with bioinformatic analysis has emerged as an essential tool to acquire relevant knowledge about cellular signaling pathways (24). Thereby, transcriptome analysis of bronchoalveolar lavage (BAL) cells, which reflects immune and pathological changes in the lung, would be an accurate approach to reveal dynamic changes in the host cellular responses against viral infection, shedding new light on PRRSV-1 immunopathogenesis at lung level.

Previous studies have explored changes in RNA-seq profile upon PRRSV infection using only one strain, mainly PRRSV-2, and determining these changes by means of microarrays, which have weaknesses relying on existing knowledge about the genome sequence (25, 26). Unlike *in vivo* studies, *in vitro* approaches present serious difficulties in identifying how the host response interacts with PRRSV, given the impossibility of connecting different cell subpopulations and cellular microenvironments (27–30). Relatively few studies have evaluated RNA-seq changes at tissue level (24), and besides, most of them conducted the analysis at 1 or 2 time points in a single tissue after PRRSV infection, frequently lymphoid tissues, which are not the main target organ for PRRSV (31, 32).

Therefore, the present study aims to explore the mechanisms involved in the modulation of host immune response at lung level, performing a time-series analysis upon experimental infection with two PRRSV-1 strains of different virulence, low-virulent 3249 strain and virulent Lena strain, identifying those terms that are conserved or strain specific in the early stages of the infection.

## RESULTS

### Lena-infected piglets exhibited hyperthermia and marked clinical signs associated with severe interstitial pneumonia, acute suppurative bronchopneumonia, and the highest lung viral load.

**FIG 1:**
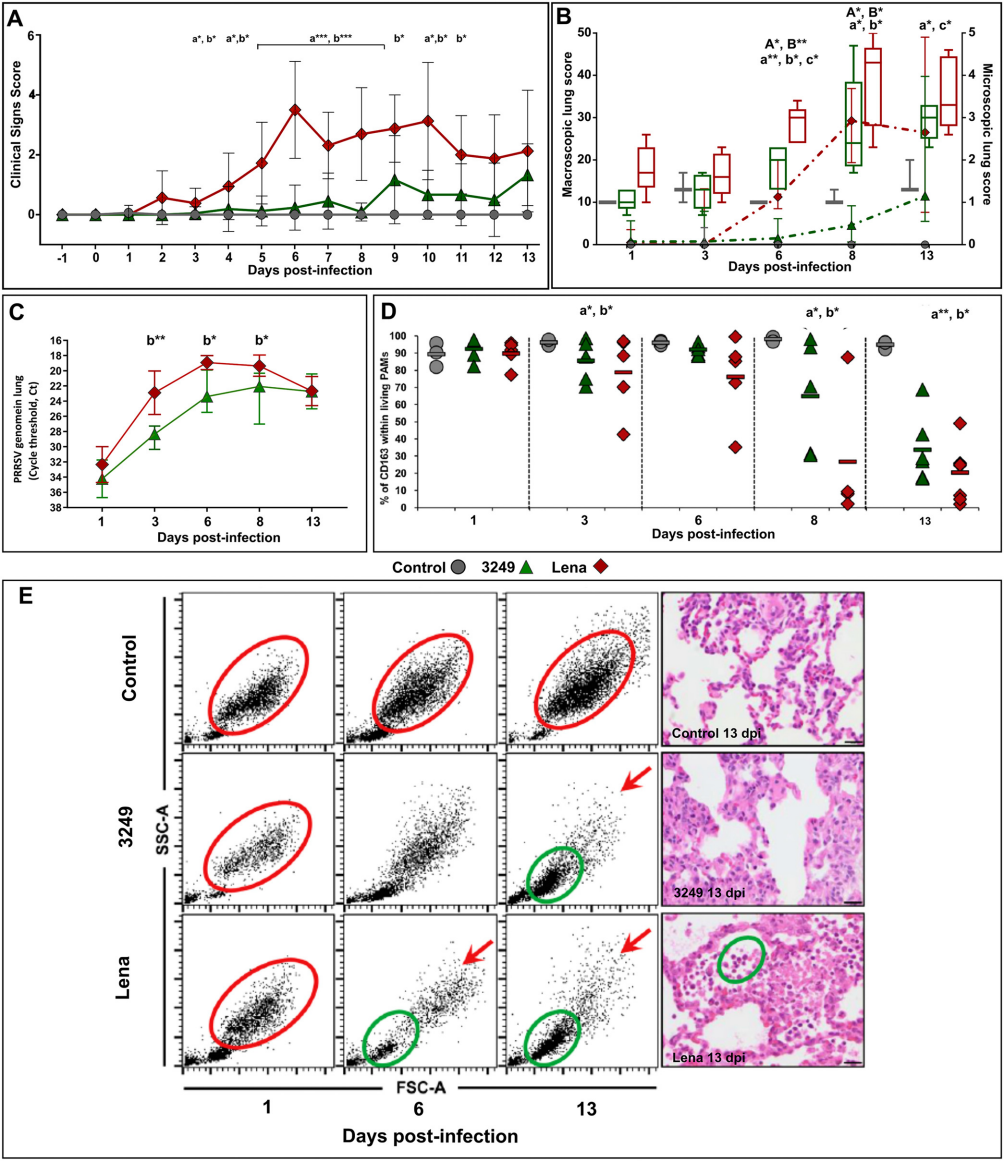
PRRSV lung viral load was quantified by RT-qPCR (A). Viral load is represented by changes in the quantification cycle (Cq) (control, gray circles; 3249, green triangles; Lena, red diamonds). Frequency of live CD163^+^ PAMs (B). Freshly isolated BAL cells from control and PRRSV-1-infected pigs were stained and analyzed for the expression of CD163 by FCM. The scatter dot plot shows the frequency of CD163^+^ cells in control, 3249, and Lena group along the experimental infection. Changes in BAL cells subpopulation by FCM (FSC-A versus SSC-A) according to histopathological findings from a representative pig of the control, 3249, and Lena infected group at 1, 6, and 13 dpi (C). Red circles indicate living potential PAMs according to light scatter properties (size and granularity). Red arrows show the decrease of the above-mentioned subset in 3249- and Lena-infected pigs. Green circles indicate a mixture of neutrophils, monocytes, and, to a lesser extent, lymphocytes, according to light scatter properties. Microscopic pictures for each representative animal at 13 dpi supporting FCM findings. Bars, 20 μm. Statistical differences between groups are indicated (*, *P < *0.05; **, *P < *0.01).

Gross lesions and histopathology were thoroughly described by Rodríguez-Gómez et al. (40). In brief, PRRSV-1-infected piglets exhibited clinical signs associated with respiratory disease. Furthermore, virulent Lena-infected pigs presented hyperthermia for a long period (mean above 40.5°C) with marked clinical signs, which reached the maximum at 6 dpi. At necropsy, lung macroscopic lesion score increased gradually throughout the study in both PRRSV-1-infected groups, due to severe interstitial pneumonia and getting to the highest score in the Lena group for the additional presence of extensive areas of pulmonary consolidation in cranial and middle lobes from 6 dpi onwards, which was confirmed by histopathological evaluation. Virulent Lena strain induced an earlier (2 out of 5 piglets PRRSV-1 positive at 1 dpi) and higher replication in the lung compared to low-virulent 3249 strain (*P < *0.01 at 3 dpi; *P < *0.05 at 6 and 8 dpi), displaying a lung viral load peak at 6 (Cq 18.9 ± 0.9) and 8 dpi (Cq 22.9 ± 2.5), respectively (Fig. 1A). Control animals did not show clinical signs and minimal lung lesion, remaining as RT-qPCR-negative for PRRSV-1 throughout the study.

### CD163^+^ cells decreased within live PAMs together with a mixed influx of neutrophils, monocytes, and lymphocytes in BAL cells of Lena-infected pigs.

FCM analysis in BAL cells were thoroughly described by Rodríguez-Gómez et al. (2019). Briefly, CD163 expression was studied only in live BAL cells from either control or PRRSV-infected pigs. Control animals showed a homogeneous and stable subset of cells within live PAMs, compatible with PAMs because of size and granularity properties as well as CD163 labeling, throughout the study (Fig. 1B, red circle, and Fig. 1C). By contrast, both subsets, PAMs and CD163^+^ cells, decreased alongside the study in PRRSV-1-infected pigs (Fig. 1B, red arrows, and 1C). This drop was more marked and occurred earlier (3 dpi) in Lena-infected pigs than in 3249 group (8 dpi) and was accompanied by a mixture of neutrophils, monocytes, and, to a lesser extent, lymphocytes (Fig. 1B, green circle) from 6 dpi onwards in Lena-infected pigs and at 13 dpi in 3249-infected animals.

### Time-series differential gene expression pattern related to PRRSV-1 infection.

**FIG 2:**
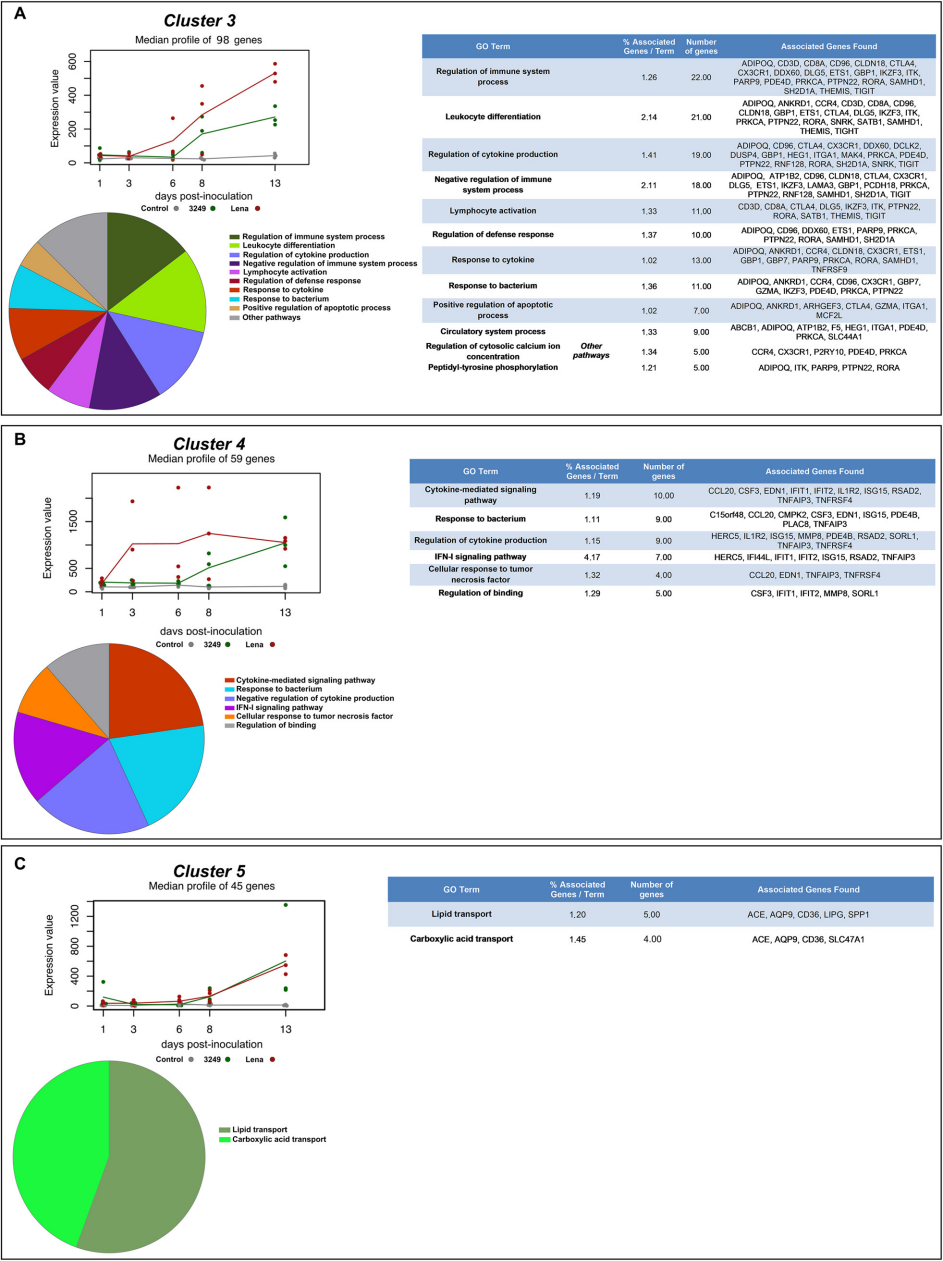
MaSigPro analysis of RNA-seq time-series data set. Clusters 3 (A), 4 (B), and 5 (C) showed distinct temporal profiles associated with low-virulent 3249 and virulent Lena strain infection. The median expression of all genes in each cluster was plotted for control (gray), 3249 (green), and Lena strain (red) along the different time points. Solid lines depicting the median and solid plots show the individual value. Gene Ontology (GO) analysis of clusters 3 (A), 4 (B), and 5 (C). ClueGO and CluePedia were used to conduct a functional enrichment analysis. Tables list the top terms of GO biological processes (BPs) and immune system processes (ISPs) associated with genes grouped in each cluster over time. Overview pie chart shows the proportion of genes associated with the top functional groups.

A total of 1,935 and 8,352 significant variable genes were detected by MasigPro during the time course for 3249- and Lena-infected pigs, respectively. After clustering, these significant variable genes were subdivided into 5 different clusters according to their expression profiles, thus, the genes included within a cluster presented a similar expression pattern. Fig. 2 illustrates the median expression level of clusters 3, 4, and 5 at each time point. Clusters 1 and 2 showed an inconsistent trend among experimental groups. Cluster 3 consisted of 98 genes whose median expression level increased in 3249-infected piglets from 8 dpi throughout the study, but this increase was higher and earlier, from 6 dpi, in Lena-infected piglets. GO enrichment-functional analyses were performed to gain biological understanding of variable genes included in cluster 3, which were mainly enriched in GO involved in “regulation of immune system process,” “leukocyte differentiation,” “regulation of cytokine production,” “negative regulation of immune system process,” “lymphocyte activation,” “response to cytokine,” and “positive regulation of apoptotic process” (Fig. 2A). Genes in cluster 4, a total of 59, followed a similar kinetics to the one described above for cluster 3 in 3249-infected piglets, but for Lena-infected pigs, the expression of these genes had a marked and rapid increase from 1 to 3 dpi remaining roughly constant throughout the study (Fig. 2). The significantly enriched GO terms related to cluster 4 were “cytokine-mediated signaling pathway,” “response to bacterium,” “regulation of cytokine production,” “IFN-I signaling pathway,” and “cellular response to tumor necrosis factor” (genes and pathways are listed in Fig. 2B). Cluster 5 comprised 45 genes whose expression rose from 8 to 13 dpi in both infected groups (Fig. 2). Genes in cluster 5 were involved in the GO terms “lipid transport” and “carboxylic acid transport” (Fig. 2C).

### DEGs profile in BAL cells of PRRSV-1-infected piglets.

**FIG 3:**
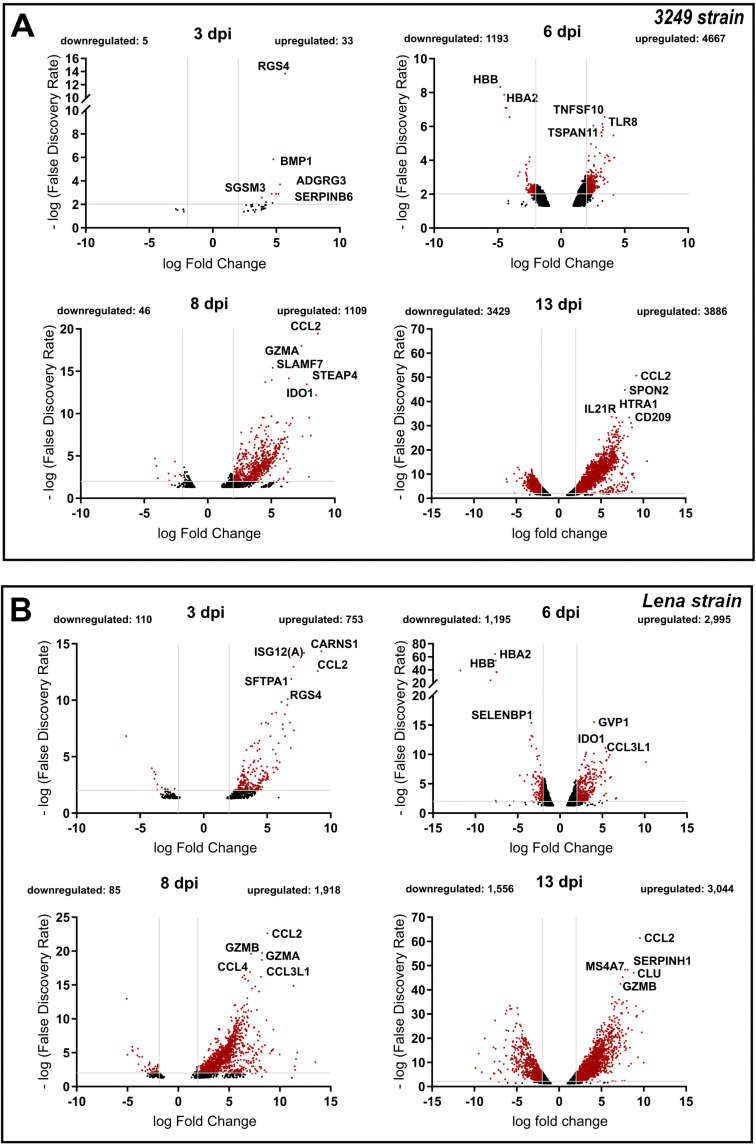
DEGs in 3249- (A) and Lena-infected (B) piglets MLN compared to non-infected control piglets. Volcano plots illustrate DEGs in 3249- (A) and Lena-infected (B) piglets to non-infected control piglets at different time points (3, 6, 8, and 13 dpi). Red dots show DEGs with an FDR* *<* *0.05 and an absolute log_2_ fold change ≥ 1, underlining the top 5 DEGs with a higher fold change. Because of the low number of DEGs not data was showed at 1 dpi.

DEGs were identified in response to 3249 or Lena infection by comparing the gene expression levels of PRRSV-1-infected animals to control animals at each time point using an *FDR *<* *0.05 and a log2FC ≥ 1.0 (upregulated genes) or log2FC ≤ –1.0 (downregulated genes) as the cut-off criteria. Thus, a total of 14,368 DEGs in 3249 data set and 11,656 DEGs in Lena data set were found to be differentially expressed along the study (Fig. 3A and B).

Similar dynamic changes in the gene expression level were observed in both 3249- and Lena-infected piglets, with an increasing number of DEGs along the study. As expected, in both infected groups, the lowest number of DEGs was observed at 1 dpi (data not showed). At 3 dpi, 38 DEGs (downregulated: 5; upregulated: 33) and 863 DEGs (downregulated: 110; upregulated: 753) were found in 3249- and Lena-infected piglets, respectively; and at 6 dpi, 5,860 DEGs (downregulated: 1,193; upregulated: 4,667) and 4,190 DEGs (downregulated: 1,195; upregulated: 2,995) were identified. At 8 dpi, a lower number of DEGs was observed probably due to cell death phenomena, with 1,155 DEGs (downregulated: 46; upregulated: 1,109) for 3249 and 2,003 DEGs (downregulated: 85; upregulated: 1918) for Lena. The highest number of DEGs were found at 13 dpi with 7,315 genes (downregulated: 3,886; upregulated: 3,429) and 4,600 genes (downregulated: 1,556; upregulated: 3,044) differentially expressed for 3249 and Lena groups, respectively (Fig. 3A and B).

### Identification and functional classification of conserved DEGs in response to 3249 strain infection.

**FIG 4:**
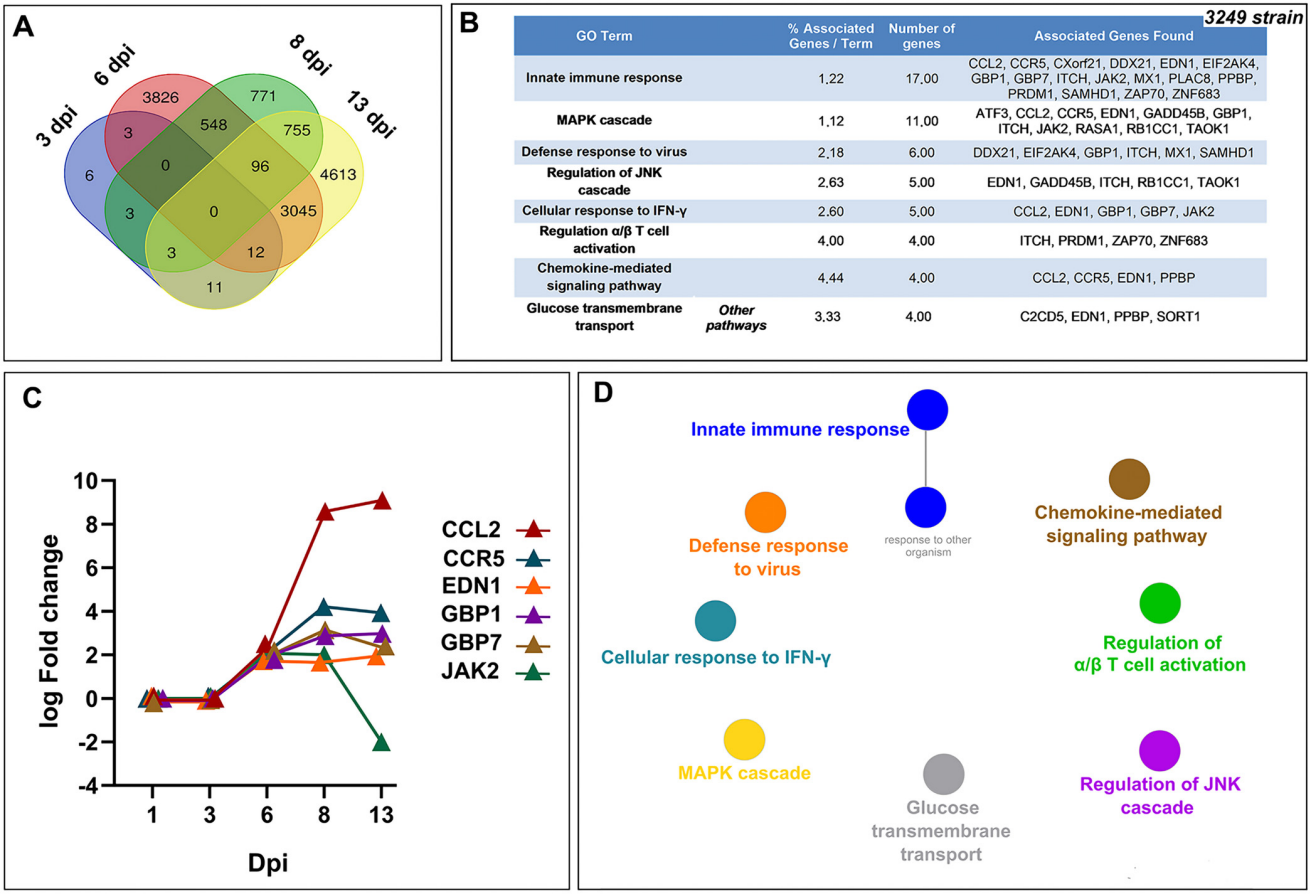
Venn diagram displaying the distribution of DEGs in low-virulent 3249-infected piglets at each time point (A). Gene ontology (GO) analysis of 96 overlapped DEGs in response to 3249 strain infection at 6–8–13 dpi. Table lists the top terms of GO biological processes (BPs) and immune system processes (ISPs) enriched with 96 overlapped DEGs (B). Pattern of expression of representative genes (*CCL2*, *CCR5*, *EDN1*, *GBP1*, *GBP7*, *JAK2*) for the most relevant pathways (C). Functional network of BPs and ISPs pathways for the module were visualized in Cytoscape with ClueGo and CluePedia (D). Only the statistically significant terms (FDR* *<* *0.05) in each group are represented. Terms are displayed as nodes (filled circle) linked by edges (lines) based on their kappa value (≥ 0.4), where only the label of the most significant term per group is shown.

A total of 14,368 DEGs were identified in response to 3249 infection during the study. Most of the DEGs (9,218) were expressed at only one of the evaluated time points, hence, we focused on those DEGs that were conserved in two or more time points, to achieve a better understanding of the infection. Thus, whereas no DEGs were concomitantly identified during the early time points of infection (1 or 3 dpi), 96 overlapped DEGs were found at 6–8–13 dpi, of which 13 genes were downregulated and 83 were upregulated (Fig. 4A). To gain further insight into the potential functions of these 96 DEGs, a functional-enrichment analysis based on the GO database was performed to explore BPs and ISPs. The significantly enriched GO terms with a *P value* < 0.05 are illustrated in Fig. 4B to D. The results of the GO analysis reported that most DEGs were mainly involved in the “innate immune response” (17 DEGs, 28.80%), “MAPK cascade” (11 DEGs, 18.64%), “defense response to virus” (6 DEGs, 7.55%), “regulation of JNK cascade” (5 DEGs, 8.47%), “cellular response to interferon γ (IFN-γ)” (5 DEGs, 8.47%), and “regulation of α/β T-cell activation” (4 DEGs, 6.78%), among others. The patterns of expression of representative genes for low-virulent 3249 strain are illustrated in Fig. 4C. Fig. 4B-4D list the symbol and number of genes enriched in each GO term.

**FIG 5:**
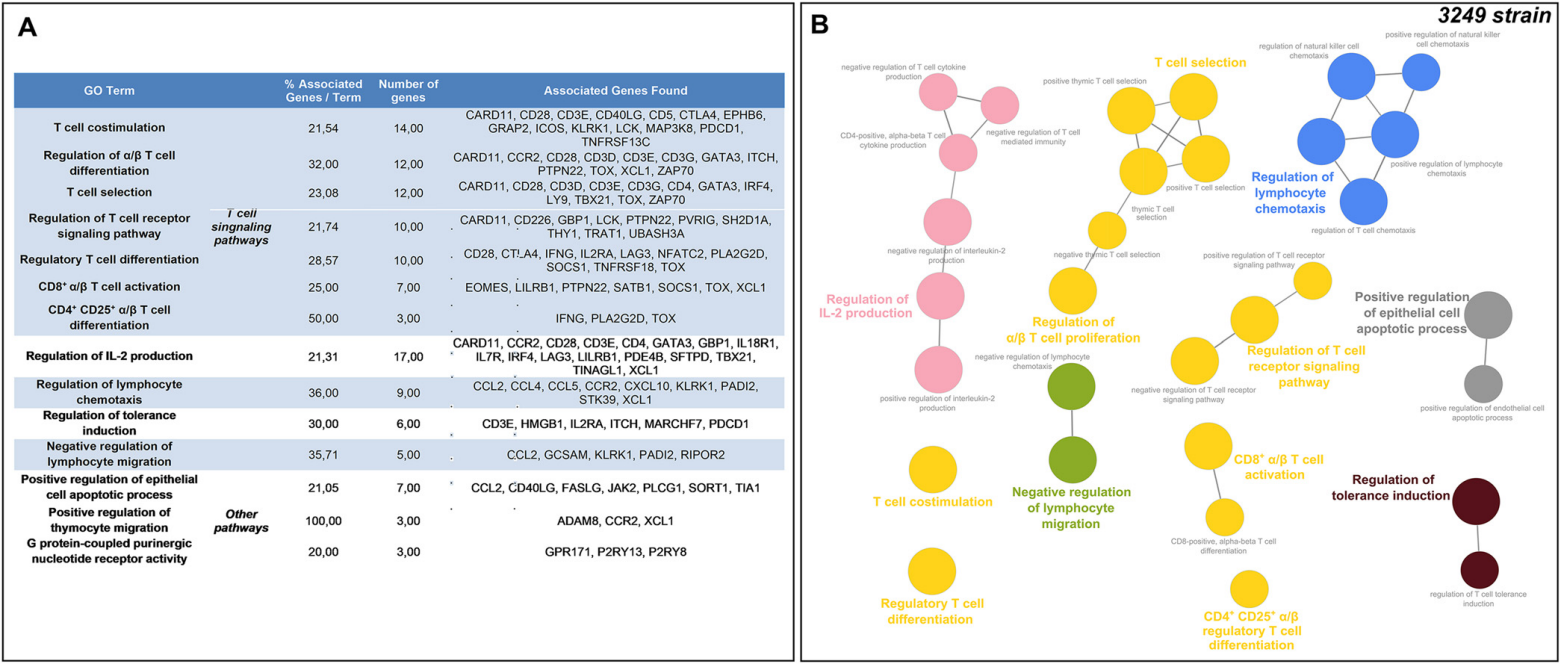
Gene Ontology (GO) analysis of 854 overlapped DEGs in response to 3249 strain infection at 8–13 dpi. Table lists the top terms of GO biological processes (BPs) and immune system processes (ISPs) enriched with 854 overlapped DEGs (A). Functional network of BPs and ISPs pathways for the module were visualized in Cytoscape with ClueGo and CluePedia (B). Only the statistically significant terms (FDR* *<* *0.05) in each group are represented. Terms are displayed as nodes (filled circle) linked by edges (lines) based on their kappa value (≥ 0.4), where only the label of the most significant term per group is shown.

Moreover, the number of conserved DEGs increased at 8–13 dpi, with 854 DEGs identified at these time points (Fig. 4A). Among these genes, only 16 genes were downregulated, whereas 838 were upregulated. In this case, the GO functional-enrichment analysis (BPs and ISPs categories) revealed that T-cell-related pathways were significantly altered with 7 different GO terms (“T-cell co-stimulation,” “regulation of α/β T-cell differentiation,” “T-cell selection,” “regulation of T-cell receptor signaling pathway,” “regulatory T-cell differentiation,” “CD8^+^ α/β T-cell activation,” and “CD4^+^CD25^+^ α/β T-cell differentiation”) and an occurrence of enriched genes over 50%. In addition, other highly enriched GO terms were related to “regulation of interleukin-2 (IL-2) production” (17 DEGs, 14.41%), “regulation of lymphocyte chemotaxis” (9 DEGs, 7.63%), or “regulation of tolerance induction” (6 DEGs, 5.08%). Fig. 5A and B list the symbol and number of genes enriched in each GO term.

### PPI network construction and Hub genes identification for 3249 infection.

**FIG 6:**
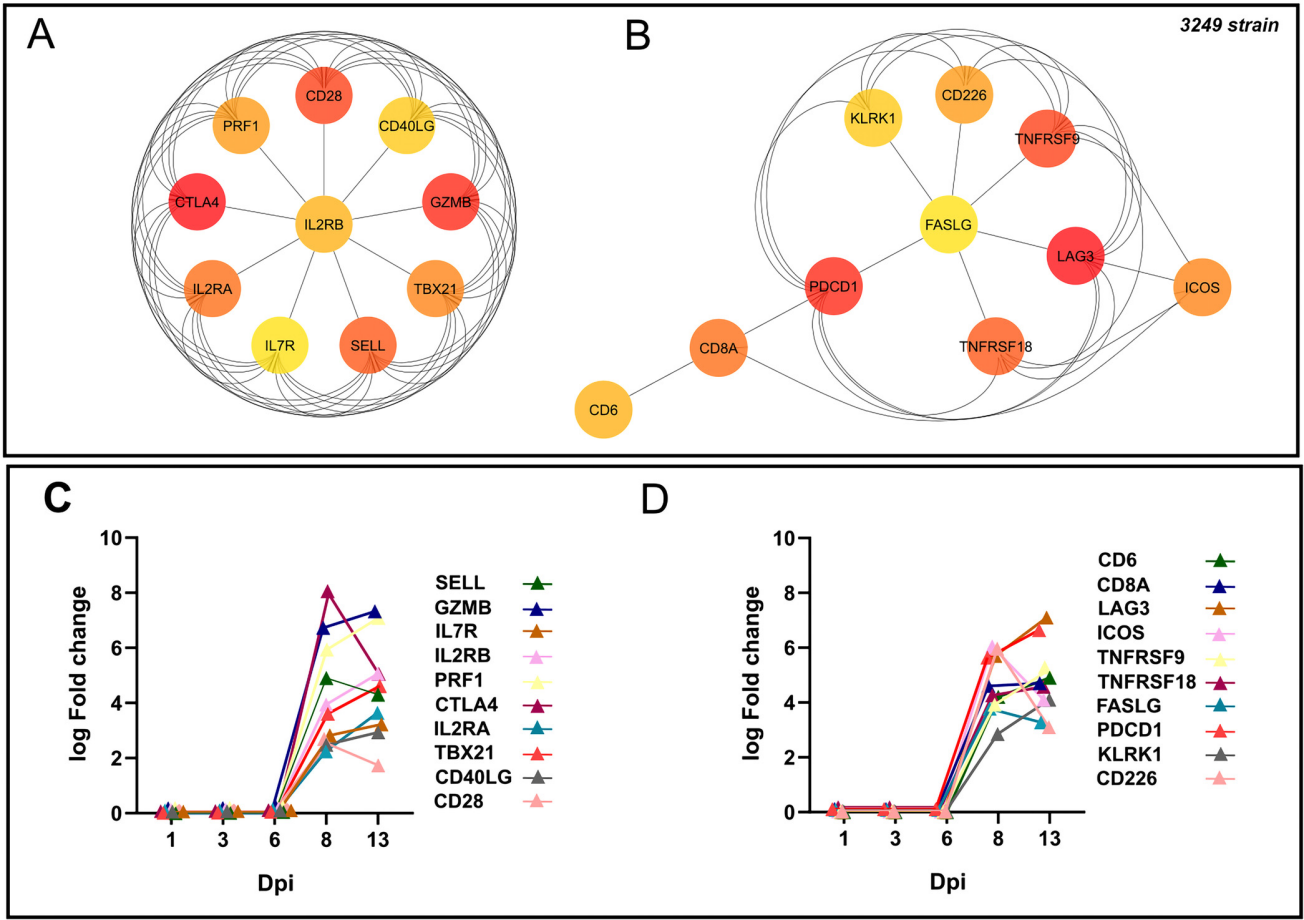
Hub genes network for low-virulent 3249 strain. Hub genes for 3249 strain were disclosed according to Maximal Clique Centrality (MCC) (A), and Density of Maximum Neighborhood Component (DMNC) (B) algorithms were identified from the whole PPI network. Kinetic of expression of Hub genes for 3249 strain along the infection (C and D). The fold change for each Hub gene was illustrated as the median of the group at 1, 3, 6, 8, and 13 dpi.

**FIG 7:**
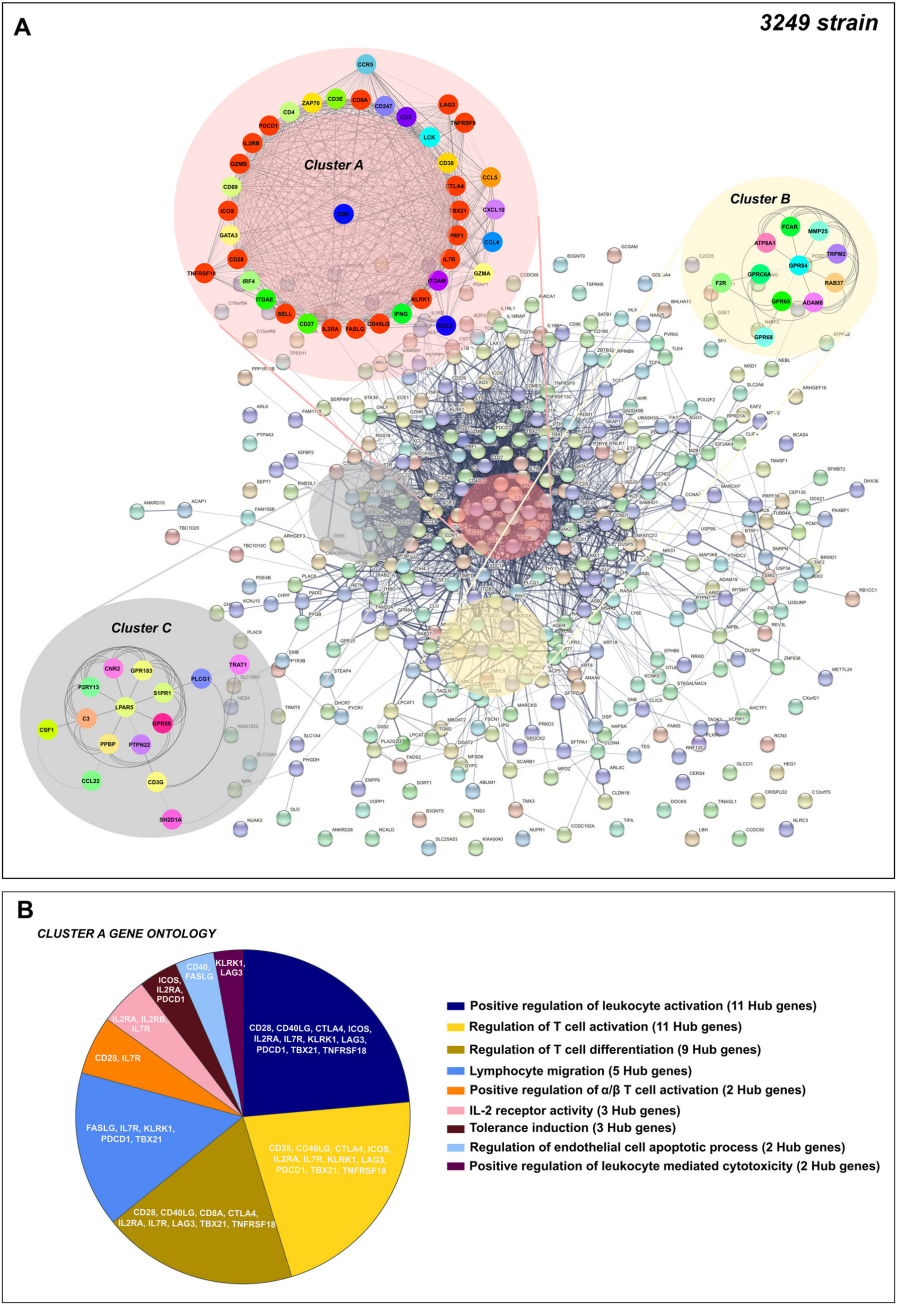
PPI network of 854 overlapped DEGs in response to 3249 strain infection at 8–13 dpi (A). Network was constructed by STRING database and visualized by Cytoscape, underlining the significant clusters A, B, and C (κ-core > 6), which were identified by means of MCODE. The genes calculated by Maximal Clique Centrality (MCC) and Density of Maximum Neighborhood Component (DMNC) algorithms were selected as Hub genes (genes with the highest degree of connectivity) by CytoHubba plugin in Cytoscape. Most of the Hub genes (red color) were included in cluster 1. GO enrichment analysis (biological processes, BPs, and immune system processes, ISPs, categories) of DEGs included within cluster A (B). Overview pie chart shows the proportion of genes associated with the top functional groups, indicating the name of Hub genes in each term. Table 1 lists the top terms of GO (BPs and ISPs).

The PPI network was constructed with the 854 conserved DEGs found at 8 and 13 dpi by the STRING database, with 402 nodes and 2,400 edges. The Hub genes were selected from the whole PPI network using the MCC and DMNC algorithm of the CytoHubba plugin. According to the MCC and DMNC scores, the top 10 highest-scored genes found for each approach include *PDCD1*, *LAG3*, *TNFRSF18*, *ICOS*, *CD8A*, *CD6*, *CTLA4*, *CD28*, *GZMB*, *TBX21*, *SELL*, *IL7R*, *IL2RA*, *IL2RB*, *PRF1*, *FASLG*, *KLRK1*, *CD226* and *CD40LG* (Fig. 6A and B). Also, the kinetic of expression of these genes is illustrated in Fig. 6C–6D. Because of the complexity, the whole PPI network was analyzed using the MCODE plugin to identify essential PPI network modules, making the understanding of molecular mechanisms more approachable. The top 3 significant clusters with a k-core > 6 were named as A, B, and C, respectively, and selected as sub-networks (Fig. 7A). Interestingly, all the Hub genes identified for the 3249 strain were included in cluster A. “Positive regulation of leukocyte activation” (11 out of 20 Hub genes), “regulation of T-cell activation (11 out of 20 Hub genes) and differentiation (9 out of 20 Hub genes),” “lymphocyte migration” (5 out of 20 Hub genes), “positive regulation of α/β T-cell activation” (2 out of 20 Hub genes), “IL-2 receptor activity” (3 out of 20 Hub genes), and “tolerance induction” (3 out of 20 Hub genes) were the main terms involved after screening of GO enrichment analyses (BPs and ISPs categories) of genes included within cluster A (Fig. 7B, Table 1).

**TABLE 1:** Gene Ontology (GO) analysis of Cluster A DEGs in response to 3249 strain infection at 8–13 dpi

GO term	% associated genes/term	No. of genes	Associated genes found
Positive regulation of leukocyte activation	5.01	25,00	CCL2, CCL5, CD2, CD27, CD28, CD38, CD3E, CD4, CD40LG, CD5, CTLA4, GATA3, ICOS, IFNG, IL2RA, IL7R, IRF4, ITGAM, KLRK1, LAG3, LCK, PDCD1, TBX21, TNFRSF18, ZAP70
Regulation of T cell activation	6.55	23,00	CCL2, CCL5, CD2, CD27, CD28, CD3E, CD4, CD40LG, CD5, CTLA4, GATA3, ICOS, IFNG, IL2RA, IL7R, IRF4, KLRK1, LAG3, LCK, PDCD1, TBX21, TNFRSF18, ZAP70
Regulation of T cell differentiation	8.50	20,00	CCL5, CD2, CD247, CD27, CD28, CD3E, CD4, CD40LG, CD8A, CTLA4, GATA3, IFNG, IL2RA, IL7R, IRF4, LAG3, LCK, TBX21, TNFRSF18, ZAP70
Lymphocyte migration	6.61	16,00	CCL2, CCL4, CCL5, CCR5, CD27, CD3E, CD4, CXCL10, FASLG, GATA3, IFNG, IL7R, KLRK1, PDCD1, TBX21, ZAP70
Positive regulation of α/β T cell activation	6.06	6,00	CD28, CD3E, GATA3, IFNG, IL7R, ZAP70
IL-2 receptor activity	66.67	5,00	CCR5, CD4, IL2RA, IL2RB, IL7R
Tolerance induction	14.29	4,00	CD3E, ICOS, IL2RA, PDCD1
Regulation of endothelial cell apoptotic process	7.84	4,00	CCL2, CD40LG, FASLG, GATA3
Positive regulation of leukocyte mediated cytotoxicity	5.08	3,00	ITGAM, KLRK1, LAG3

### Identification and functional classification of conserved DEGs in response to virulent Lena strain infection.

**FIG 8:**
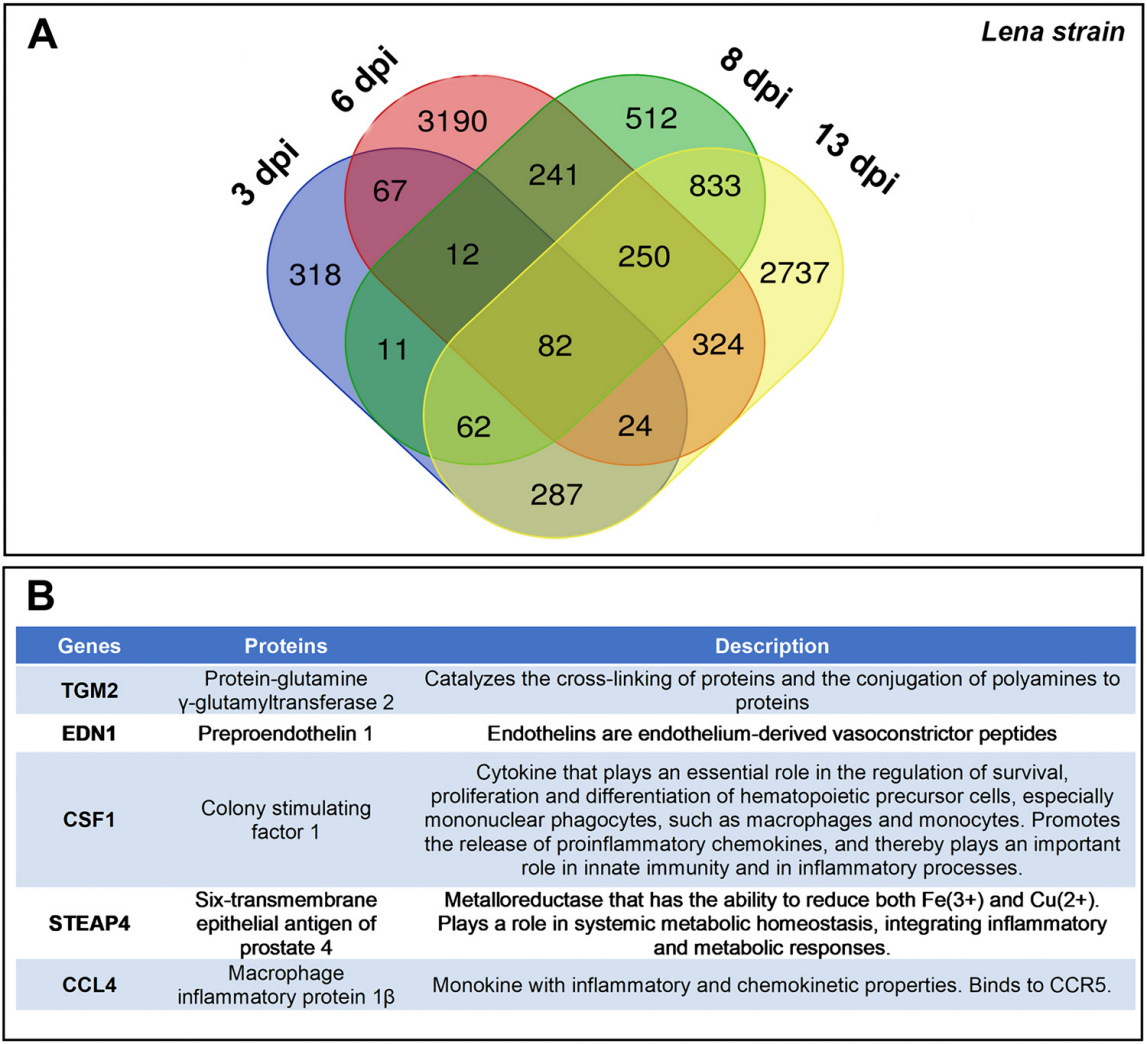
Table lists 5 upregulated DEGs conserved in response to virulent Lena infection at 1–3–6–8–13 dpi (A). Venn diagram displaying the distribution of DEGs in virulent Lena-infected piglets at each time point (B).

A sum of 11,656 DEGs were found in the Lena data set (Fig. 8A), with most of them (6,760 genes) being expressed only at one single time point during the infection. The same approach described above was followed to analyze the 3249 data set; the analysis was addressed in DEGs identified at two or more time points.

Noteworthy, 5 conserved DEGs, all of them upregulated, were identified at 1–3–6–8–13 dpi including colony-stimulating factor 1 (*CSF1*), chemokine (C-C motif) ligands 4 (*CCL4*), six-transmembrane epithelial antigen of prostate 4 (*STEAP4*), transglutaminase 2 (*TGM2*), and endothelin 1 (*EDN1*) (Fig. 8B).

**FIG 9:**
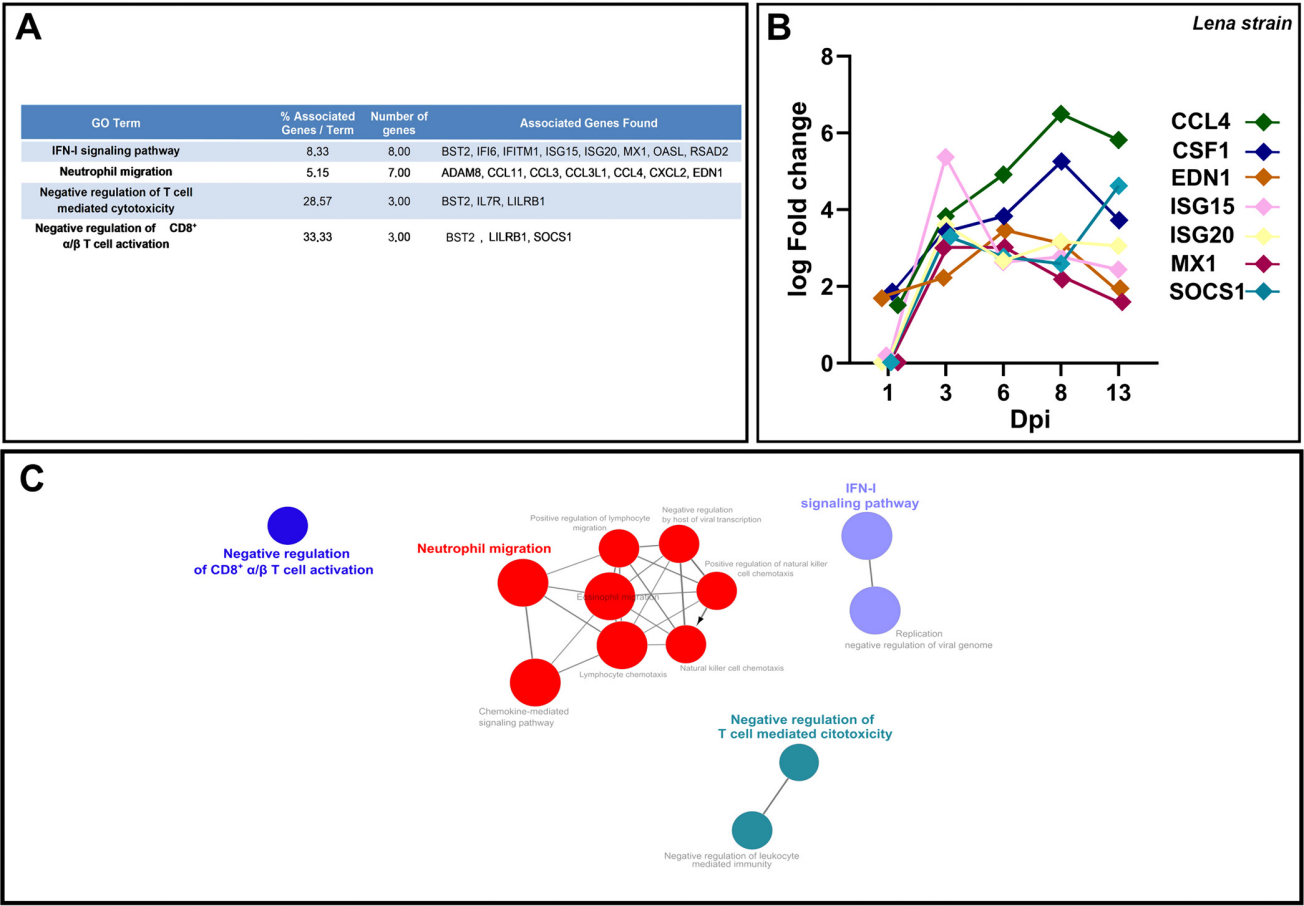
Gene Ontology (GO) analysis of 82 overlapped DEGs in response to Lena strain infection at 3–6–8–13 dpi. Table lists the top terms of GO biological processes (BPs) and immune system processes (ISPs) enriched with 82 overlapped DEGs (A). Pattern of expression of representative genes (*CCL4*, *CSF1*, *EDN1*, *ISG15*, *ISG20*, *MX1*, *SOCS1*) for the most relevant pathways (B). Functional network of BPs and ISPs pathways for the module were visualized in Cytoscape with ClueGo and CluePedia (C). Only the statistically significant terms (FDR* *<* *0.05) in each group are represented. Terms are displayed as nodes (filled circle) linked by edges (lines) based on their kappa value (≥ 0.4), where only the label of the most significant term per group is shown.

At 3–6–8–13 dpi, 82 overlapped DEGs were identified among which only 3 were downregulated, whereas 79 were upregulated (Fig. 8A). In the next step, a GO functional-enrichment analysis was conducted (BPs and ISPs categories), with most of the DEGs enriched in terms associated with “IFN-I signaling pathway” (8 DEGs, 19.05%), “neutrophil migration” (7 DEGs, 16.78%), “negative regulation of T-cell mediated cytotoxicity” (3 DEGs, 7.14%) and “negative regulation of CD8^+^ α/β T cell activation” (3 DEGs, 33.33%). Fig. 9 lists the symbol and number of genes enriched in each GO term, as well as the patterns of expression of representative genes for the virulent Lena strain (Fig. 9B).

**FIG 10:**
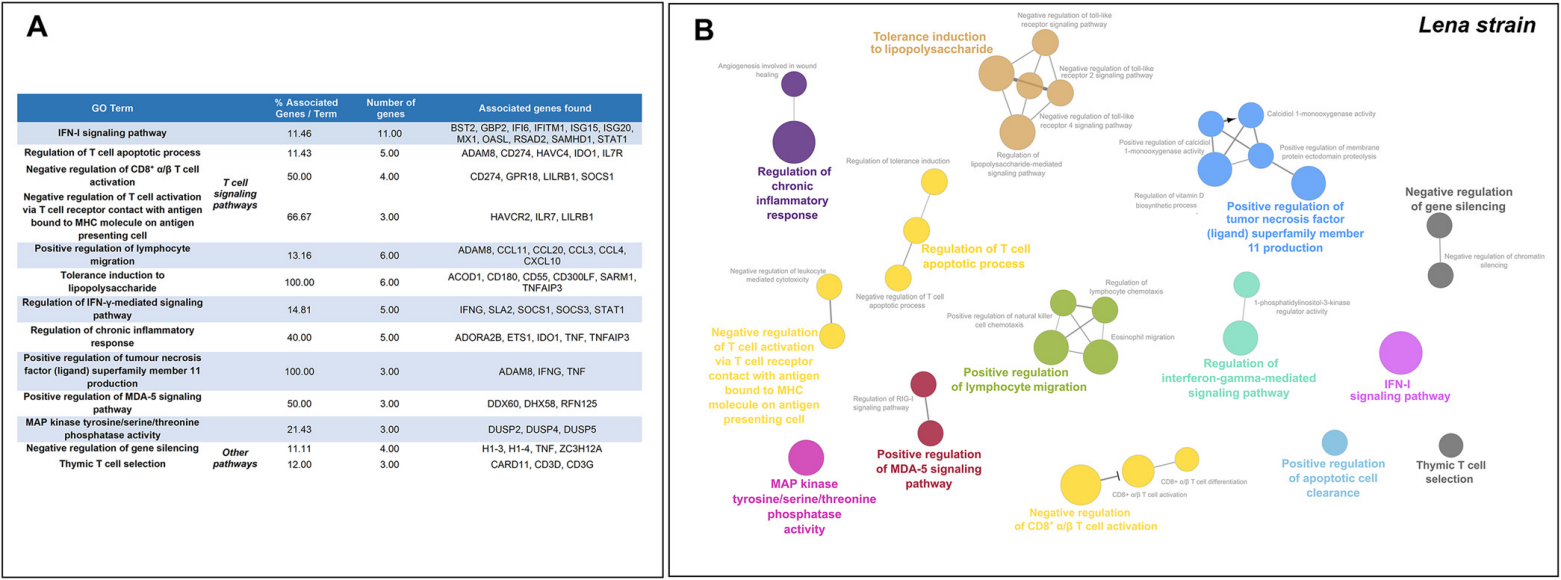
Gene Ontology (GO) analysis of 332 overlapped DEGs in response to Lena strain infection at 6–8–13 dpi. Table lists the top terms of GO biological processes (BPs) and immune system processes (ISPs) enriched with 332 overlapped DEGs (A). Functional network of BPs and ISPs pathways for the module was visualized in Cytoscape with ClueGo and CluePedia (B). Only the statistically significant terms (FDR* *<* *0.05) in each group are represented. Terms are displayed as nodes (filled circle) linked by edges (lines) based on their kappa value (≥ 0.4), where only the label of the most significant term per group is shown.

Consequently, a sum of 332 overlapped DEGs was disclosed at 6–8–13 dpi, most of them (311) upregulated with only 21 genes downregulated (Fig. 8B). In this case, according to the GO functional-enrichment analysis (BPs and ISPs categories), the majority of DEGs were significantly enriched within “IFN-I signaling pathway” (11 DEGS, 14.67%) and T-cell signaling pathways (11 DEGs, 16.10%) (Fig. 10). Moreover, other highly enriched GO terms were identified, underlining “positive regulation of lymphocyte migration” (6 DEGs, 8.00%), “tolerance induction to lipopolysaccharide” (6 DEGs, 8.00%), “regulation of IFN-γ-mediated signaling pathway” (5 DEGs, 6.67%), and “regulation of chronic response” (5 DEGs, 6.67%). Fig. 10 lists the symbol and number of genes enriched in each GO term.

**FIG 11:**
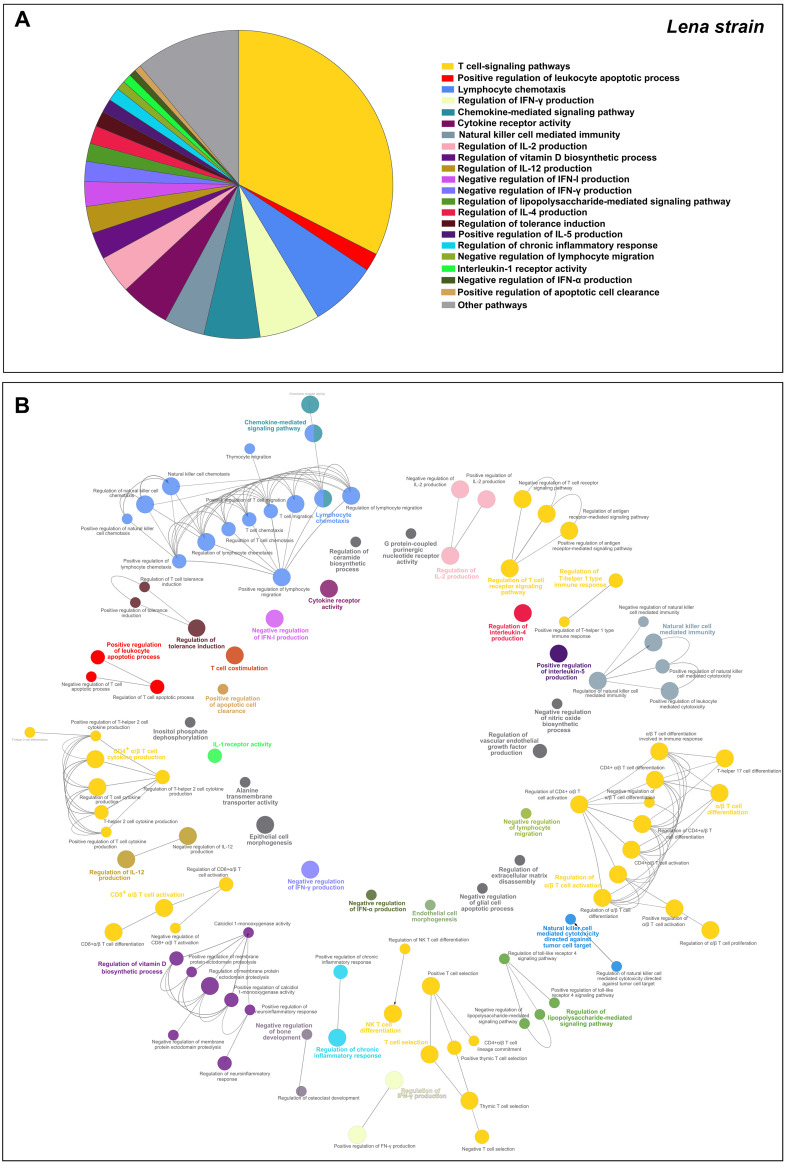
Gene Ontology (GO) analysis of 1,227 overlapped DEGs in response to Lena strain infection at 8–13 dpi. Overview pie chart illustrates the top terms of GO biological processes (BPs) and immune system processes (ISPs) enriched with 1,227 overlapped DEGs, indicating the proportion of genes associated with each term (A). Functional network of BPs and ISPs pathways for the module were visualized in Cytoscape with ClueGo and CluePedia (B). Only the statistically significant terms (FDR* *<* *0.05) in each group are represented. Terms are displayed as nodes (filled circle) linked by edges (lines) based on their kappa value (≥ 0.4), where only the label of the most significant term per group is shown. Table 2 lists the top terms of GO (BPs and ISPs).

Finally, a total of 1,227 overlapped genes were differentially expressed at 8–13 dpi, which included 48 downregulated and 1,179 upregulated genes (Fig. 8B). After screening of GO enrichment analysis, T-cell signaling pathways were found to be highly impaired, with 138 DEGs (32.43%) enriched in 9 different GO terms (Fig. 11). In addition, we found further significant enriched GO terms such as “lymphocyte chemotaxis” (30 DEGs, 6.9%), “regulation of IFN-γ production” (27 DEGs, 6.21%), “chemokine-mediated signaling pathway” (25 DEGs, 5.75%), “NK cell mediated immunity” (18 DEGs, 4.14%), “regulation of vitamin D biosynthetic process” (12 DEGS, 44.44%), “positive regulation of leukocyte apoptotic process” (8 DEGs, 2.07%), and “regulation of tolerance induction” (7 DEGs, 1.61%). Fig. 11 and Table 2 list the symbol and number of genes enriched in each GO term.

**TABLE 2:** Table lists the top terms of GO biological processes (BPs) and immune system processes (ISPs) enriched with 1,227 overlapped DEGs in response to Lena strain infection at 8–13 dpi

GO term		% associated genes/term	No. of genes	Associated genes found
***T cell signaling pathways***				
α/β T cell differentiation		25.23	37.00	AGER, 8CL118, CCR2, CD274, CD28, CD3E, CD55, CRTAM, EBl3, EOMES, GATA3, GPR18, GPR183, HLX, IFNG, IL18R1, IRF4, ITK, LY9, NFK81Z, NLRP3, PRDM1, PTPN22, RORA, RSAD2, RUNX3, SAT81, SFTPA1, SOCS1, T8 × 21, TCF7, TOX, ZAP70, ZBT816, ZC3H12A, ZFPM1, ZNF683
Regulation of α/β T cell activation		24.00	24.00	AGER, CCR2, CD274, CD28, CD3E, CD55, CRTAM, E8I3, GATA3, HLX, IFNG, IRF4, LILR81, NFK8IZ, NLRP3, PRDM1, PTPN22, RUNX3, SOCS1, T8 × 21, ZAP70, Z8T816, ZC3H12A, ZNF683
T cell selection		32.69	17.00	8CL 118, CARD11, CCR7, CD1D, CD28, CD3D, CD3E, CD3G, CD4, GATA3, IRF4, LY9, T8 × 21, THEMIS, TOX, ZAP70, ZFPM1
T cell co-stimulation		23.08	15.00	CARD11, CCR7, CD274, CD28, CD3E, CD40LG, CDS, CTLA4, EPH86, GRAP2, ICOS, KLRK1, LCK, MAP3K8, PDCD1
Regulation of T cell receptor signaling pathway		28.26	15,00	CARD11, CCR7, CD226, G8P1, GCSAM, LCK, PRKCH, PRKD2, PTPN22, PVRIG, SH2D1A, SLA2, THY1, TRAT1, U8ASH3A
CD8^+^ α/β T cell activation		35.71	10.00	CD274, CRTAM, EOMES, GPR18, LILR81, PTPN22, RUNX3, SAT81, SOCS1, TOX
CD4^+^ CD25^+^ α/β T cell cytokine production		34.78	8.00	CD55, GATA3, IL18R1, IL18, NLRP3, RSAD2, T8 × 21, TINAGL1
Regulation of T-helper type immune response		21.43	6.00	CCR2, HAVCR2, HLX, IL 18R1, IL18, IL1 RL1
NK T cell differentiation		50.00	6.00	ITK, PRDM1, SFTPA1, TOX, Z8T816, ZNF683
Lymphocyte chemotaxis		24.24	30.00	ADAM8, CCL11, CCL2, CCL20, CCL22, CCL3L 1, CCL4, CCL5, CCR2, CCR5, CCR7, CMKLR1, CXCL 10, CXCL 13, CXCL 14, CXCL2, CXCR3, CXCR6, EDN1, GCSAM, GPR75, GPR183, ITG87, KLRK1, PP8P, RIPOR2, S1PR1, STK39, ZAP70, XCR1
Regulation of IFN-y production		24.11	27.00	CCR2, CCR7, CD2, CD226, CD274, CD3E, CD96, CRTAM, E8I3, GATA3, HAVCR2, IL 10, IL 12R82, IL 18R1, IL18, IL1RL 1, INH8A, ISG15, KLRK1, LILR81, PDE48, PDE40, PTPN22, RASGRP1, TNF, ZC3H12A, ZFPM1
Chemokine-mediated signaling pathway		23.33	25.00	ADAM8, CCL 11, CCL2, CCL20, CCL22, CCL3L 1, CCL4, CCL5, CCR2, CCR5, CCR7, CMKLR1, CXCL 10, CXCL 13, CXCL 14, CXCL2, CXCR3, CXCR6, EDN1, GPR75, GPR183, KLRK1, PP8P, STK39, XCR1
Cytokine receptor activity		22.00	22.00	CCR2, CCR5, CCR7, CD4, CMKLR1, CRLF2, CXCR3, CXCR6, E8I3, GPR75, IL12R82, IL15RA, IL18R1, IL18RAP, IL1R2, IL1RAP, IL1RL1, IL21R, IL2RA, IL2R8, IL7R, XCR1
Natural killer cell mediated immunity		20.00	18.00	CD1A, CD1 D, CD226, CD96, CRTAM, GZM8, HAVCR2, KLRK1, F2RL1, ITGAM, LAG3, LILR81, RA827A, RASGRP1, SERPIN89, SH2D1A, SLAMF7, TINAGL 1
Regulation of IL-2 production		27.87	17.00	CARD11, CCR2, CD28, CD3E, CD4, GATA3, G8P1, HAVCR2, IL18, IRF4, LAG3, PDE48, PDE4O, PRKD2, SFTPD, T8 × 21, TNFAIP3, MEFV, TIGIT
Regulation of vitamin D biosynthetic process		44.00	12.00	ADAM8, CST7, IFNG, IL 10, IL 18, MMP8, NUPR1, SPHK1, SNAI1, TNF, TIMP1, TIMP3
Regulation of IL-12 production		20.00	12.00	ACP5, AGER, CCR7, CD40LG, CMKLR1, 1001, IFNG, IL 10, LILR81, LT8,
Negative regulation of IFN-I production		23.40	11.00	ACOD1, DHX58, HAVCR2, HERC5, IL 10, ISG15, LILR81, NLRC3, RNF125, TNFAIP3, U8E2L6
Negative regulation of IFN-γ production		22.50	9.00	CD274, CD96, GATA3, HAVCR2, IL 10, IL 1RL 1, INH8A, LILR81, ZC3H12A
Positive regulation of leukocyte apoptotic process		23.53	8.00	ADAM8, CCL5, CD27, CD274, IDO1, IL10, NR4A3, PDCD1
Regulation of lipopolysaccharide-mediated signaling pathway		20.00	8.00	ACOD1, CD180, CD55, F2RL 1, LTF, PTPN22, PRKCA, TNFAIP3
Regulation of IL-4 production		25.81	8.00	CD28, CD3E, CD40LG, GATA3, HAVCR2, IRF4, NLRP3, ZFPM1
Regulation of tolerance induction		35.00	7.00	CD274, CD3E, HAVCR2, IDO1, IL2RA, MARCHF7, PDCD1
Positive regulation of IL-5 production		46.15	6.00	CRLF2, GATA3, IL 1RAP, IL 1RL 1, NLRP3, PDE4O
Regulation of chronic inflammatory response		60.00	6.00	ADORA28, CCL5, IDO1, IL 10, TNF, TNFAIP3
Negative regulation of lymphocyte migration		28.67	4.00	CCL2, GCSAM, KLRK1, RIPOR2
lnterleukin-1 receptor activity		57.14	4.00	IL18R1, IL 1 R2, IL 1 RAP, IL 1 RL 1
Negative regulation of IFN-α production		42.86	3.00	HAVCR2, IL 10, NLRC3
Positive regulation of apoptotic cell clearance		33.33	3.00	C3, C4A, CCL2
Epithelial cell morphogenesis		21.95	9.00	8CL 118, CCDC88C, CLDN3, COL 18A1, FAT1, FLN8, HEG1, HRH2, PECAM1
***Other pathways***				
Regulation of vascular endothelial growth factor production		21.21	7.00	ADORA28, C3, CCR2, HPSE, IL 18, RORA, SULF2
Regulation of extracellular matrix disassembly		21.05	4.00	CARMIL2, ETS1, FSCN1, PDPN
G protein-coupled purinergic nucleotide receptor activity		26.67	4.00	P2RY10, P2RY13, P2RY6, P2RY8
lnositol phosphate dephosphorylation		25.00	4.00	INPP1, INPP48, INPP58, SYNJ2

### PPI network construction and Hub genes identification for virulent Lena infection.

**FIG 12:**
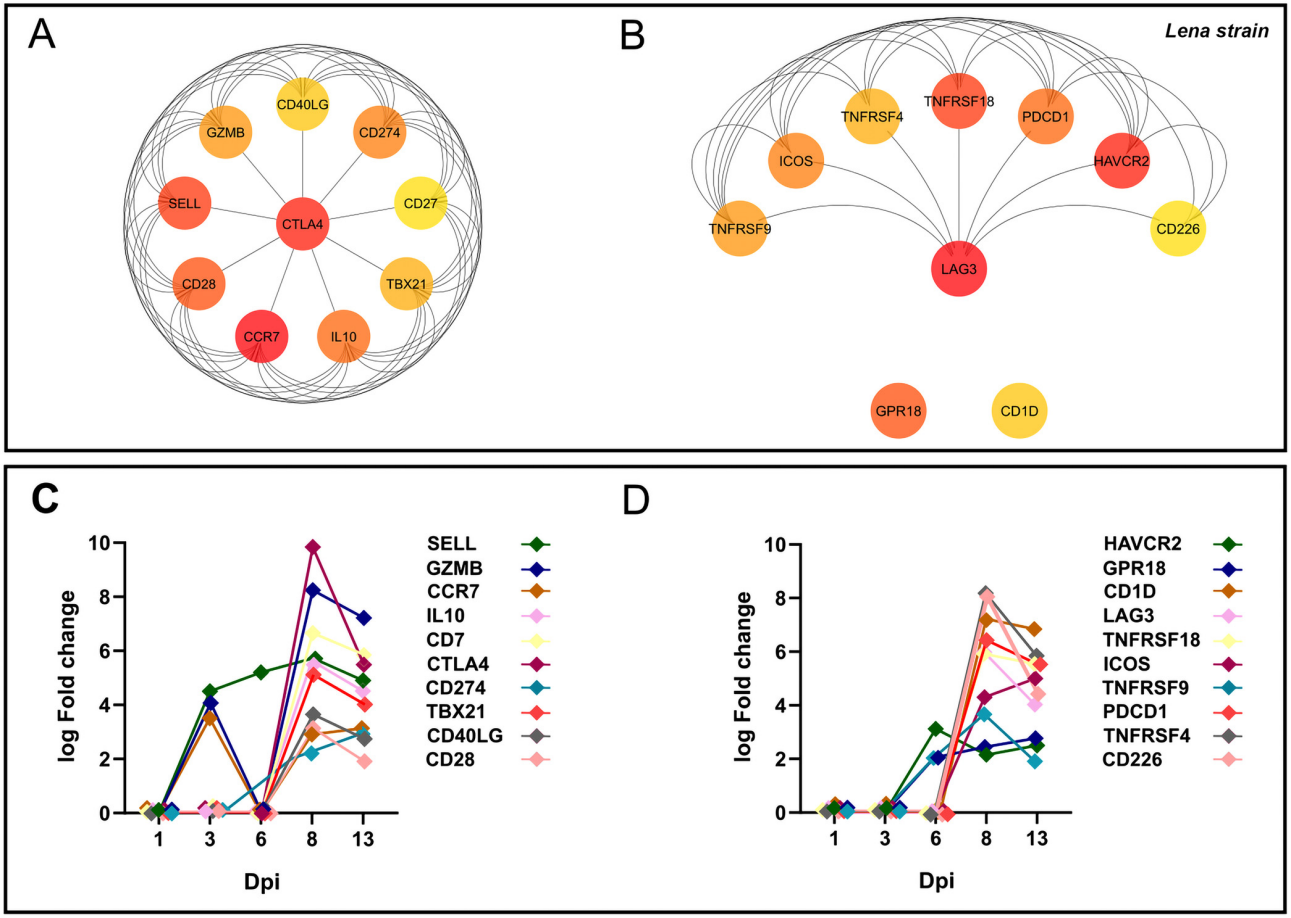
Hub genes network for virulent Lena strain. Hub genes for Lena strain were disclosed according to Maximal Clique Centrality (MCC) (A), and Density of Maximum Neighborhood Component (DMNC) (B) algorithms were identified from the whole PPI network. Kinetic of expression of Hub genes for Lena strain along the infection (C and D). The fold change for each Hub gene was illustrated as the median of the group at 1, 3, 6, 8, and 13 dpi.

**FIG 13:**
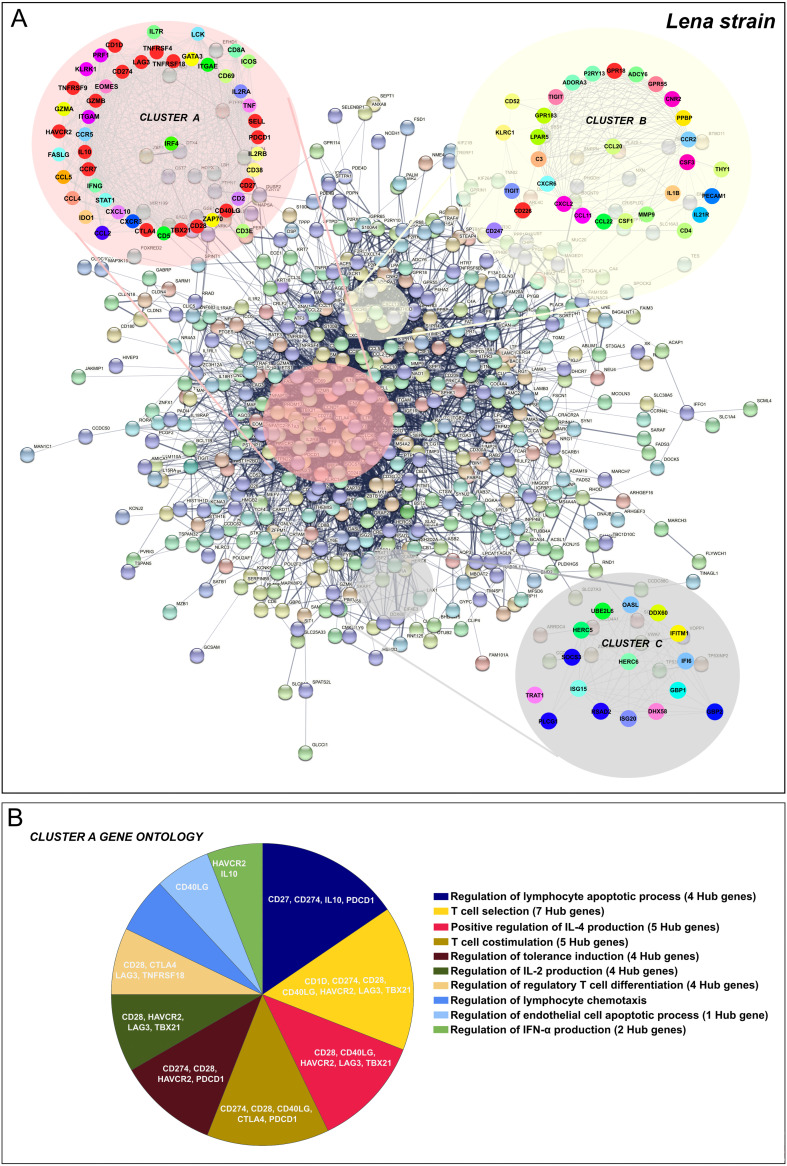
PPI network of 1,227 overlapped DEGs in response to Lena strain infection at 8–13 dpi (A). Network was constructed by STRING database and visualized by Cytoscape, underlining the significant clusters A, B, and C (k-core > 6), which were identified by means of MCODE. The genes calculated by Maximal Clique Centrality (MCC) and Density of Maximum Neighborhood Component (DMNC) algorithms were selected as Hub genes (genes with the highest degree of connectivity) by CytoHubba plugin in Cytoscape. Most of the Hub genes (red color) were included in cluster A. GO enrichment analysis (BPs and ISPs categories) of DEGs included within cluster A (B). Overview pie chart showing the proportion of genes associated with the top functional groups, indicating the name of Hub genes in each term. Table 3 lists the top terms of GO (BPs and ISPs).

In this case, the 1,227 overlapped DEGs detected at 8–13 dpi were utilized to construct the PPI network by the STRING database, finding 560 nodes and 4,461 edges. Afterwards, according to the MCC and DMNC algorithm of the CytoHubba plugin, the top 10 highest-scored genes found for each approach from the whole PPI network were identified as Lena strain Hub genes, highlighting *CTLA4*, *CCR7*, *SELL*, *CD28*, *CD274*, *IL-10*, *GZMB*, *TBX21*, *CD40LG*, *CD27*, *LAG3*, *PDCD1*, *HAVCR2*, *ICOS*, *TNFRSF18*, *TNFRSF4*, *TNFRSF9*, *GPR18*, *CD1D* and *CD226* (Fig. 12A and B). The kinetics of expression Hub genes disclosed for the virulent Lena strain was displayed in Fig. 12C and D. Of note, several of these Lena strain Hub genes (11 out of 20) were previously identified for the 3249 strain. After analyzing the whole PPI network by MCODE plugin, the top 3 more significant sub-networks were selected and named as cluster A, cluster B, and cluster C (Fig. 13A). Cluster A, which included most of Lena strain Hub genes, was subsequently subjected to a GO functional-enrichment analysis (BPs and ISPs categories). “Regulation of lymphocyte apoptotic process” (4 out of 20 Hub genes), “T-cell selection” (7 out of 20 Hub genes), “positive regulation of IL-4 production” (5 out of 20 Hub genes), “T-cell co-stimulation” (5 out of 20 Hub genes), “regulation of tolerance induction” (4 out of 20 Hub genes), “regulation of IL-2 production” (4 out of 20 Hub genes), “regulation of regulatory T-cell differentiation” (4 out of 20 Hub genes), “regulation of lymphocyte chemotaxis,” and “regulation of IFN-α production” (2 out of 20 Hub genes) were reported as highly enriched GO terms (Fig. 13B, Table 3).

**TABLE 3:** Gene Ontology (GO) analysis of Cluster A DEGs in response to Lena strain infection at 8–13 dpi

GO term	% associated genes/term	No. of genes	Associated genes found
Regulation of lymphocyte apoptotic process	12.50	13,00	CCL5, CCR7, CCR5, CXCR3, CD27, CD274, CD3E, IDO1, IL10, IL7R, IFNG, PDCD1, TNF
T cell selection	15.38	13,00	CCR7, CD1D, CD274, CD28, CD3E, CD40LG, IDO1, GATA3, HAVCR2, IRF4, LAG3, TBX21, ZAP70
Positive regulation of IL-4 production	25.00	10,00	CD28, CD3E, CD40LG, GATA3, HAVCR2, IRF4, LAG3, TBX21, STAT1, ZAP70
T cell co-stimulation	17.74	11,00	CCR7, CD274, CD28, CD3E, CD40LG, CD5, CTLA4, ICOS, KLRK1, LCK, PDCD1
Regulation of tolerance induction	26.32	9,00	CCL5, CD274, CD28, CD3E, HAVCR2, IDO1, IL2RA, PDCD1, ZAP70
Regulation of IL-2 production	11.29	7,00	CD28, CD3E, GATA3, HAVCR2, IRF4, LAG3, TBX21
Regulation of regulatory T cell differentiation	18.18	6,00	CD28, CTLA4, IFNG, IL2RA, LAG3, TNFRSF18
Regulation of lymphocyte chemotaxis	20.00	5,00	CCL2, CCL4, CCL5, CXCL10, KLRK1
Regulation of endothelial cell apoptotic process	10.20	5,00	CCL2, CD40LG, FASLG, GATA3, TNF
Regulation of IFN-α production	10.00	3,00	HACVR2, IL10, STAT1
IL-2 receptor activity	66.67	2,00	IL2RA, IL2RB

### Verification of the DEGs expression patterns and Hub genes by RT-qPCR.

Twelve Hub genes (*CCR7*, *CD274* (PDL-1), *CD28*, *CTLA4*, *GZMB*, *HACVR2* (TIM3), *IL-10*, *LAG3*, *SELL*, *TBX21* (T-BET), *PDCD1* (PD-1), *TNFRSF9* (CD137)) found either in the Lena or 3249 group were arbitrarily selected to confirm their differential expression levels by RT-qPCR. As shown in Table 4, the qPCR results of the Hub genes agreed with those of RNA-seq analysis, validating the RNA-seq data herein reported.

**TABLE 4:** Validation of RNA-seq gene expression patterns and Hub genes using RT-qPCR (log_2_ fold change)^*a*^

Gene	Quantification method	Fold increase (log_2_)	
		3249 strain	Lena strain
*CD274* (PD-L1)	RNAseq	2.7	4.1
	qPCR	4.3	6.7
*CTLA4*	RNAseq	8.1	10.1
	qPCR	5.7	8.2
*HACVR2* (TIM3)	RNAseq	-	2.2
	qPCR	-	5.1
*LAG3*	RNAseq	5.8	7.3
	qPCR	5.7	7.5
*PDCD1* (PD-1)	RNAseq	5.3	6.4
	qPCR	5.7	7.5
*IL10*	RNAseq	-	5.6
	qPCR	-	7.3
*TBX21* (T-bet)	RNAseq	3.6	5.1
	qPCR	4.1	7.1
*CD28*	RNAseq	2.4	3.2
	qPCR	3.9	6.5
*TNFRSF9*	RNAseq	3.9	5.9
	qPCR	3.9	6.9
*GZMB*	RNAseq	6.7	8.2
	qPCR	7.0	8.7
*SELL*	RNAseq	4.8	5.6
	qPCR	4.4	6.0
*CCR7*	RNAseq	-	2.8
	qPCR	-	3.1

a Dashes indicate not found as Hub gene for 3249 strain.

## DISCUSSION

PRRSV have evolved different mechanisms to evade the host’s immune response (18, 20), favoring also secondary infections that result in severe disease such as PRDC (23). Understanding the mechanisms involved in the dysregulation of the immune response at lung level is a cornerstone for unraveling the immunopathogenesis of PRRS, one of the most economically devastating diseases for swine production.

### Time-series analysis pointed out an early and sustained upregulation of co-inhibitory receptors related to T-cell and NK cell functions over time points.

In the present study, RNA-seq was used to perform a time course analysis in BAL cells upon PRRSV *in vivo* infection with strains of different virulence to gain further insight into the potential pathways involved in modulating the host’s immune response. Clusters 3, 4, and 5 consisted of genes whose expression levels increased during the time course, although with different patterns and intensity for each strain. Genes in cluster 3, which exhibited a similar kinetics to lung lesion, were enriched in the GO terms “regulation of immune system process,” “leukocyte differentiation,” “regulation of cytokine production,” “lymphocyte activation,” and “response to cytokine and positive regulation of apoptotic process,” among others. These terms related to viral infection and immune response signaling pathways were upregulated earlier and higher in virulent Lena-infected than in low-virulent 3249-infected piglets, which may explain the severe lung injury observed. In addition, a group of genes was enriched for “negative regulation of immune system process” (Fig. 2A), including *CD96*, *CTLA4*, *RFN128*, *TIGIT*, and *SAMHD1*, which play an inhibitory role in T-cell and NK cell functions (33–38), potentially hampering viral clearance and the onset of an effective adaptive immune response.

Cluster 4 included genes that were strongly and promptly upregulated in virulent Lena-infected compared with low-virulent 3249-infected piglets. GO terms such as “cytokine-mediated signaling pathway,” “response to bacterium,” “regulation of cytokine production,” “IFN-I signaling pathway,” and “cellular response to tumor necrosis factor” were enriched in this cluster. According to our previous results and those from others, these inflammatory-related pathways contribute to the severe clinical outcomes and lung lesions observed in Lena-infected piglets, but also to an early upregulation of pro-inflammatory cytokines associated with a high viral load and the induction of regulated cell death (14, 39–42). These results confirm that the dynamics and extent of virulent Lena strain replication are critical for the disease outcome, inducing a strong and early inflammatory response in the lung.

On the other hand, cluster 5 genes involved the GO terms “lipid transport” and “carboxylic acid transport,” whose expression increased in parallel in both PRRSV-1-infected groups from 8 to 13 dpi. Viruses are able to exploit cellular lipid transport, signaling, and metabolism for their own advantage, making viral entry, replication, or assembly of virions easier (43). DEGs associated with lipid signaling pathways have already been described during PRRSV infection, suggesting that PRRSV strains could modify host’s cells lipid metabolism to enable either viral replication or virion release (24, 26).

### The interplay among IFN-I/IFN-II orchestrated an antiviral response resulting in IFN-stimulated genes unable to control efficiently viral replication, probably due to PRRSV countermeasures.

IFN-I leads to a signal transduction cascade of hundreds of IFN-stimulated genes (ISGs), a powerful instrument that interferes with viral replication (44, 45). In our case, a screen of ISGs, enriched in IFN-I signaling pathway, was upregulated in BAL cells from both virulent Lena-infected (*BST2*, *IFI6*, *IFITM1*, *ISG15*, *ISG20*, *MX1*, *OASL*, *RSAD2*, *STAT1*) and low-virulent 3249-infected piglets (*EIF2AK4*, *GBP1*, *MX1*) from 3 and 6 dpi onwards, respectively. Interferon regulatory factors (IRF) have been also found to be overexpressed in PAMs infected *in vitro* with Lena and Lelystad virus strains (29), as well as during the early stage of PRRSV-2 infection (24, 46, 47). Many of these upregulated genes could point to a continuous antiviral state (45, 48), since *ISG20*, *MX1*, and *RSAD2* have the capacity to inhibit viral replication (49–51), nevertheless, PRRSV has been reported to inhibit *IRF3* as well as several of these ISGs such as *STAT1*, *ISG15*, and *IFITM1* (52, 53), by viral non-structural proteins (nsp1α, nsp1β, nsp2, nsp4, and nsp11) (53–56). Thus, unlike the 3249 strain, the virulent Lena strain induced an upregulation of DEGs enriched in negative regulation of IFN-I and type II IFN (IFN-II) at 8–13 dpi in our study. Altogether, these results draw an interplay among IFN-I/IFN-II induced antiviral response and PRRSV countermeasures resulting in ISGs unable to control efficiently viral replication, leading to an incomplete viral clearance and potential persistent infection (57).

### Early upregulation of chemokines and cytokines in response to virulent Lena infection led to an acute lung injury.

Virulent PRRSV-1 strains trigger an exacerbated and early pro-inflammatory response compared with low-virulent strains, leading to severe lung injury, secondary bacterial infection, and hence, acute respiratory symptoms (12, 39, 58, 59). A considerable number of DEGs were enriched in GO terms involved in macrophages activation, pro- and anti-inflammatory cytokines, as well as chemokines for the recruitment of neutrophils, monocytes, and lymphocytes to the site of infection in both PRRSV-1-infected groups during the study. Interestingly, Lena-infected pigs showed 5 DEGs, which were upregulated from 1 to 13 dpi, with three of them (*CSF1*, *CCL4*, and *EDN1*) showing striking inflammatory properties, such as monocyte and macrophage proliferation and differentiation (*CSF1*) (60), vasoconstriction and pro-inflammatory response associated with acute lung injury (ALI) (*EDN1*) (61), or neutrophil migration (*CCL4*) (62). Although, PAMs are the key players during PRRSV infection, the GO analysis suggests that chemotaxis of neutrophils and their likely degranulation are continued over the time points (25, 46), leading to alveolar–capillary barrier damage, increased vascular permeability, and hence, a higher extent of lung damage, as has been observed in this study.

According to the dynamic changes observed in BAL cells from PRRSV-1-infected piglets, the “positive regulation of lymphocyte migration” term was observed in virulent Lena-infected piglets from 6 dpi onwards, including upregulated expression of *ADAM8*, *CCL11*, *CCL20*, *CCL3*, *CCL4*, and *CXCL10*. By contrast, in the 3249 group not only were these genes overexpressed later (8-13 dpi), but also, genes enriched in “negative regulation of lymphocyte migration” were found (*CCL2*, *GCSAM*, *KLRK1*, *PADl2*, *RIPOR2*). *CCL20/MIP-3α* is a strong chemotactic factor for lymphocytes, while a weak factor for neutrophils, that is upregulated by IFN-γ, tumor necrosis factor (TNF) and lipopolysaccharide, inducing a marked lung damage upon viral infection (63). *CCL2*, *CCL3*, *CCL4*, and *CCXL10* are produced in the lung in the early phase of lung inflammation, working as a chemotactic factor for macrophages, NK cells, and T cells during viral infection (64). In addition, there was an upregulation of genes associated with “positive regulation of apoptotic cell clearance” (*C3*, *C4*, *CD300LF*, *CCL2*) in Lena-infected piglets from 6 to 13 dpi, probably, due to the severe activation of regulated cell death phenomena at lung level induced by the virulent Lena strain (42), suggesting an attempt to remove dead cells and cellular debris to restore tissue damage.

Interestingly, many cytokine-signaling pathways were found to be regulated in Lena- compared with 3249-infected piglets, highlighting an interplay among pro- and anti-inflammatory responses that are likely to be activated in a virulence dependent fashion and hence influencing the profile of innate cytokines as well as the development of adaptive immunity. IFN-γ release pathway was observed in the 3249 group from 6 dpi onwards, whereas *SOCS1* and *SOSC3*, potent IFN-γ inhibitors (65), were early overexpressed in the Lena group (6 dpi) followed by an interface of upregulated genes that may promote IFN-γ production (8–13 dpi). *IL1B* (IL-1β) and *TNF* genes were upregulated in the Lena group, unlike the 3249 group. While classical PRRSV strains are reported to show a delayed and weak systemic production of these cytokines (66–69), this proinflammatory profile seems to be a hallmark of virulent PRRSV strains (24, 25, 39, 58, 59), resulting in being useless in controlling viral replication but exacerbating clinical signs and lung damage.

Moreover, the virulent Lena strain induced a strong regulation of adaptive-cytokines-related pathways such as “regulation of IL-2, IL-4, and IL-12 production,” as well as an upregulation of *IL-10* (IL-10) and *IL18BP* (IL-18BP, IL-18 binding protein) from 8 to 13 dpi, in contrast to the 3249 group, in which only “regulation of IL-2 production” was found. Most of the genes enriched in “regulation of IL-12 production” (7 out of 12), such as *IL-10* and *IDO1*, show a negative regulation of this pathway. Likewise, upregulation of *IL-10* expression has been reported to impair Th1 response during PRRSV infection, inducing a niche for viral-specific regulatory T cells (Tregs) in a strain-dependent fashion, though Tregs induction after PRRSV infection is controversial (70–72). Although no changes in the serum concentration of IL-10 were observed in Lena- and 3249-infected pigs (41), IL-10 could be locally secreted in the lung, exerting a paracrine *in situ* effect. *IL18BP* gene encodes a soluble receptor that hijacks IL-18, blocking the engagement of IL-18 to its receptor, which in turn inhibits IL-18-induced IFN-γ production and alters Th1 response (73, 74). The anti-inflammatory mediators herein reported may play a double-edged sword role, limiting the inflammatory response during the early stage of the infection but also, when sustained in time, weakening the host’s immune response against PRRSV and hence impairing viral clearance.

### Activation of T-cell signaling pathways was overlapped in virulent and low-virulent PRRSV-1 strains in a time-dependent manner.

Activation of T-cells is crucial for anti-PRRSV adaptive immunity since T cells are key players in tackling viral infection, particularly, during the first stages of infection (17, 75). The virulent Lena strain caused a prompt upregulation of genes enriched in “negative regulation of T cell mediated cytotoxicity,” “negative regulation of CD8^+^ α/β T-cell activation,” “negative regulation of T-cell activation via T-cell receptor contact with antigen bound to MHC molecules on antigen presenting cells,” and “regulation of T cell apoptotic process,” which would impair the induction of CD8^+^ cytotoxic T lymphocytes (CTLs) needed to eliminate virus-infected cells, release of pro-inflammatory cytokines, and establish memory cells (18, 75). Besides, genes associated with “regulation of chronic inflammatory response” were found to be overexpressed, suggesting that virulent Lena strain would be able to overcome the host’s antiviral strategies, inducing a persistent infection. Thus, many of these DEGs (*SOCS1*, *LILRB1*(CD85J), *CD274* (PD-L1), *HAVCR2* (TIM3), and *IDO1*) enriched in the above-mentioned pathways are immune checkpoints associated with other persistent viral infections inducing T-cell exhaustion (33, 76, 77). On the other hand, a substantial number of genes were regulated from 8 dpi onwards in either Lena- or 3249-infected groups, and these DEGs were enriched in quite similar T-cell-related pathways, highlighting “T-cell co-stimulation,” “T-cell selection,” “regulation of α/β T-cell differentiation,” and “CD8^+^ α/β T-cell activation.” T-cell stimulation is an essential mechanism that relies on co-stimulatory and co-inhibitory receptors to achieve an effective T-cell activation (78). Several viruses involved in persistent infections exploit these adaptive mechanisms to evade immune-mediated viral clearance, leading to viral persistence and, hence, a functionally inferior T-cell response (33, 79). Thereby, either Lena or 3249 elicited an upregulation of co-stimulatory receptors (*CD28*, *ICOS*, *CD40LG* (CD154), *CD27*, *TNFRSF4* (OX40), *TNFRSF9* (CD137) or *TNFRSF18* (GITR)) but also inhibitory receptors (*CD5*, *CTLA4*, *CD274* (PD-L1)/*PDCD1*, *HAVCR2* (TIM3), *LAG3*, *TIGIT*, *TOX*), some of which were also overexpressed in the thymus of these animals (80), suggesting an attempt to hamper T-cell activation. The transcription factors *GATA3* and *TBX21* (T-bet), overexpressed in both low-virulent 3249 and virulent Lena infected groups, have been reported to be involved in T-cell maturation and Th1 polarization of porcine alpha-beta T-cells (81); however, *EOMES*, upregulated as well, and *TBX21* could also cooperate to sustain exhausted CD8^+^ T-cell subsets (78, 82, 83). Besides, we found a profile of upregulated DEGs enriched in “regulation of chronic inflammatory response” (*ADORA2B*, *CCL5*, *1001*, *IL-10*, *TNF*, *TNFAIP3*) and “regulation of tolerance induction” (*CD274*, *CD3E*, *HAVCR2*, *1001*, *IL2RA*, *MARCHF7*, *PDCD1*). Considering all the above-mentioned, two different scenarios could be drawn. First, these pathways regulate self-tolerance, minimizing bystander tissue damage and hindering the inflammatory response observed mainly in Lena-infected piglets. A second possibility is that PRRSV might induce T-cell anergy if upregulation of inhibitory receptors is sustained over time and associated with a high level of viral antigen, as it has been described for other arterivirus, such as Equine arteritis virus (84). Although multiple elements are responsible for T-cell exhaustion (79), these findings, together with the well-known persistence of PRRSV in lymphoid organs (57), point out that PRRSV-1 could induce adaptive immune tolerance, making viral transmission in the farm easier.

### Immune checkpoints were revealed as Hub genes conserved in both virulent and low-virulent PRRSV-1 infection.

A Hub gene analysis was conducted by CytoHubba, since Hub genes set up more complex interactions compared with other genes in a PPI network and hence play a central role in the mechanisms of disease (85). Consequently, exploring Hub genes and their associated key pathways would be essential for a better understanding of PRRSV-1 infection at lung level. Eleven of 20 Hub genes were shared in both 3249 (Fig. 6) and Lena (Fig. 12) strains. Most of the Hub genes followed a similar kinetic for both strains, Lena and 3249, increasing their expression from 8 to 13 dpi. Nevertheless, several of them, SELL, *GZMB*, and *CCR7* (at 3 dpi), and *HAVCR2* (TIM-3), *TNFRSF9* (CD137), and *GPR18* (at 6 dpi) were promptly upregulated for the virulent Lena strain, probably associated with the marked clinical signs, the earlier and stronger peak of replication, and severe pneumonia. Remarkably, 13 of 20 Hub genes are considered as co-stimulatory immune checkpoints that are gaining a special interest nowadays, because of their role in cancer and chronic viral infections (86, 87). Mainly the Lena, but also the 3249 strain, induced an upregulation of co-stimulatory receptors such as *CD28*, *ICOS*, and several members of the TNF receptor family (*CD40LG* (CD154), *CD27*, *TNFRSF4* (OX40), *TNFRSF9* (CD137) and *TNFRSF18* (GITR)), which are considered as key players in CD4^+^ and CD8^+^ T-cell differentiation and expansion in acute and chronic viral infections (88–90). By contrast, co-inhibitory receptors, such as *CTLA4*, *CD274* (PD-L1), *PDCD1* (PD-1), *LAG3*, and *HAVCR2* (TIM-3), working together with IL-10, have been reported in acute viral infection, such as SARS-CoV-2 or influenza virus, playing a double role, one limiting inflammation induced tissue damage and ALI, as can particularly be the case for the virulent Lena strain, and another one, inducing a progressive dysfunction of T cells, impairing viral clearance and leading to chronic infection (33, 77, 79). A sustained upregulation of these Hub genes has been closely associated with CD8^+^ and CD4^+^ T-cells dysfunction during persistent viral infections together with high viral loads and antigen levels (33, 79, 87). Subsequently, a MCODE analysis was conducted to evaluate the interaction of Hub genes with other DEGs, clustering most of them in cluster A in both Lena- and 3249-infected groups. Therefore, “regulation of lymphocyte apoptotic process,” “T-cell selection,” “positive regulation of IL-4 production,” “T-cell co-stimulation,” and “regulation of tolerance induction” seem to be significant pathways related to virulent Lena infection, whereas “positive regulation of leukocyte activation,” “regulation of T-cell activation and differentiation,” “lymphocyte migration,” and “tolerance induction” would be the essential ones for the low-virulent 3249 strain. These results highlight that the mechanism of PRRSV infection with strains of different virulence could follow common pathways with a greater activation of pro- and anti-inflammatory response, and indeed, severe lung damage in Lena-infected piglets, probably associated with a superior replication rate (41).

## CONCLUSION

The present study dissects the panoply of molecular mechanisms that govern the immunopathogenesis of PRRSV-1 infection with strains of different virulence at lung level, involving many conserved molecular pathways for virulent Lena and low-virulent 3249 strain. These included the activation of co-inhibitory and co-stimulatory immune checkpoints, revealed as Hub genes, in the pulmonary disease, which may have an impact on activation of CD8^+^ and CD4^+^ T cells and other T-cell related-pathways. Nevertheless, virulent Lena infection resulted in more pronounced and earlier upregulation of these immune checkpoints together with the regulation of cytokine-signaling pathways, orchestrating an interplay among pro- and anti-inflammatory responses. These mechanisms try to restrict inflammation induced tissue damage and ALI, mostly in virulent Lena strain infection, but also may lead to a progressive dysfunction of T cells, impairing viral clearance and persistent infection, favoring secondary bacterial infections or viral rebound. This study highlights the pivotal role that immune checkpoints could play in responding to acute PRRSV-1 infection at the lung level, although further studies should be conducted to evaluate the functional role of immune checkpoints in chronic PRRSV infection and explore a possible T-cell exhaustion state.

## MATERIALS AND METHODS

### Porcine reproductive and respiratory syndrome virus strains.

The low-virulent 3249 strain (subtype 1 PRRSV-1) was isolated from the serum of a piglet with pneumonia from a PRRSV-positive herd located in Spain in 2005 (91). The virulent Lena strain (subtype 3 PRRSV-1), considered as the prototype of PRRSV-1 virulent strains, was isolated from the lung of weak born piglets from a PRRSV outbreak characterized by high mortality rate, reproductive failure, and respiratory disorders in a Belarusian farm in 2007 (15). Both strains were propagated in porcine alveolar macrophages (PAMs), then the viral stocks were produced from the fourth passage of each strain, titrated by means of immunoperoxidase monolayer assay and expressed as tissue culture infectious doses 50 (TCID_50_)/mL (Lena strain: 10^5.66^ TCID_50_/mL; 3249 strain: 10^5.79^ TCID_50_/mL).

### Animals and experimental design.

Animals used in this study belong to a large project carried out in order to investigate the pathogenesis of PRRSV-1 strains of different virulence (40). Briefly, 65 4-week-old piglets (Landrace × Large White crossbreed), obtained from a historically PRRSV-negative farm, were randomly assigned to three different groups and housed in separate pens in biosafety level III containment facilities (IRTA-CReSA, Cerdanyola del Vallès, Barcelona, Spain): 3249 group (*n* = 25), Lena group (*n* = 25), and control group (*n* = 15). At the beginning of the study, all piglets were ELISA and PCR-negative for porcine circovirus type 2 (PCV2), PRRSV, and Mycoplasma hyopneumoniae (92, 93).

After 1 week of acclimation, piglets were inoculated with the low-virulent 3249 strain or the virulent Lena strain (1 mL/nostril, 1 × 10^5^ TCID_50_/mL by intranasal inoculation with a mucosal atomizer; MAD Nasal Intranasal Mucosal Atomization Device, Teleflex, Alcalá de Henares, Madrid, Spain). The control group was mock-inoculated with PAMs supernatant diluted in RPMI 1640 culture medium (Thermo Fisher Scientific, Barcelona, Spain). Three piglets from each experimental group were euthanized at 1, 3, 6, 8, and 13 days postinoculation (dpi). All animal procedures were performed according to the guidelines of the European Union (Directive 2010/63/EU) and approved by the IRTA Ethics Committee and by the Catalan Autonomous Government (Project 3647; FUE-2017-00533413).

### Clinical signs, gross and histopathological lung lesion, and PRRSV lung viral load.

Rectal temperature and clinical signs (liveliness, distress, and anorexia) were daily recorded from 2 days prior to inoculation until the end of the study. Hyperthermia was considered when rectal temperature was higher than 40.5°C. The score of clinical signs, ranging from 0 to 5, was recorded as previously described (41).

At necropsy, gross lung lesions were evaluated and recorded by the same pathologist as previously described by Halbur et al. (94). Parallel samples from cranial, middle, and caudal lobes of the right lung were collected and immediately frozen at −80°C for the quantification of lung viral load or fixed in 10% neutral buffered formalin (Fisher Scientific, Ltd., Loughborough, UK) for histopathological evaluation.

The histopathological findings were blindly evaluated and graded by two different pathologists. The severity of lung lesions for the interstitial pneumonia was scored as previously described (94), whereas the score for suppurative bronchopneumonia was previously described by Rodríguez-Gómez et al. (2019). Overall, the final score for each piglet was calculated as the sum of both the interstitial pneumonia and the bronchopneumonia scores.

A lung tissue homogenate was made previously to carry out RNA isolation and purification by using TRIzol LS Reagent (Thermo Fisher Scientific) followed by NucleoSpin RNA virus columns kit according to manufacturer’s protocols (Macherey-Nagel, Düren, Germany). According to subgenomic copies of the virus, results for viral load were expressed by changes in quantification cycle (Cq), to not overestimate the number of PRRSV viral particles in the lung, as previously described (95). The ORF7 RT-PCR product from both 3249 and Lena strains was firstly precipitated in ethanol and purified using ExoSAP-ITTM (Thermo Fisher Scientific). The purified products were quantified using Nanodrop 2000 (Thermo Fisher Scientific). Serial 10-fold dilutions of 3249 or Lena ORF7 RT-PCR products (ranging from 10^8^ to 10^2^ genomic copies/mL) were used as standards to determine the limit of detection (1 copy/μL) and PCR efficiency (99%). Then, viral load for either Lena- or 3249-infected piglets was determined by RT-qPCR using VetMAX PRRSV EU/NA 2.0 kit (Thermo Fisher Scientific).

### Flow cytometry (FCM) staining and analysis.

BAL samples for FCM analysis and sequencing were collected at the time of necropsy from the left lung of each piglet (40). Freshly isolated cells from BAL were adjusted to 1.5 × 10^6^ cells per sample in a final volume of 200 μL. Then, BAL cells were stained for CD163 (clone 2A10/11, IgG1, 10 μg/mL; Bio-Rad Laboratories, S.A., Alcobendas, Madrid, Spain). After washing, a second incubation step with a fluorochrome-labeled isotype-specific secondary antibody (Alexa Fluor 647 goat anti-mouse IgG1, 7 μg/mL; Invitrogen, Carlsbad, CA, USA) in combination with Live/Dead Fixable Aqua Dead Cell Stain (Invitrogen) was performed. Following surface labeling, cells were fixed and permeabilized with methanol overnight at −20°C (VWR International, Llinars del Vallès, Barcelona, Spain). FCM analysis was performed on a FACSCanto II (BD Biosciences, NJ, USA) recording 5 × 10^5^ to 1 × 10^6^ cells per sample. By making use of FlowJo software version 10 (FLOWJO LLC, Ashland, OR, USA), cells were gated according to light scatter properties (FSC-A versus SSC-A) and subjected to doublet (FSC-H versus FSC-W and SSC-H versus SSC-W) and dead cell discrimination and further analyzed for the expression of CD163.

### BAL cells, RNA isolation, and sequencing.

RNA seq analyses were performed on representative piglets (3 animals/group/time point) over time points (*n* = 45). These animals were selected according to their clinical signs, gross and microscopic lung lesion scores, as well as viral load. Total RNA was isolated from 1.5 × 10^6^ cells per sample re-suspended with 1 ml of TRIzol reagent (Thermo Fisher Scientific), and then treated with Turbo DNA-free Kit (Thermo Fisher Scientific) to remove traces of contaminating genomic DNA (gDNA) following the manufacturer’s guidelines. RNA integrity number (RIN) was assessed in the Bioanalyzer 2100 system (Agilent Technologies, CA, USA). All RNA samples used in the present study showed absence of gDNA and RIN values over 8.5.

Library preparation and sequencing were performed at the Functional Genomics Core of the Institute for Research in Biomedicine (IRB Barcelona, Spain). The cDNA libraries were prepared with 500 ng of total RNA for each individual sample using the Illumina TruSeq Stranded mRNA Sample Prep Kit (Illumina, Inc., San Diego, CA, USA). Libraries were quantified with Qubit dsDNA HS assay (Thermo Fisher Scientific) and quality was assessed by Agilent 2100 Bioanalyzer (Agilent Technologies). Subsequently, the indexed libraries were sequenced on a HiSeq2000 device (Illumina Inc.), and paired-end reads (2 × 75 base pair) were generated. The raw RNA-seq data set was deposited at NCBI Sequence Read Archive database with the accession number PRJNA704925.

### RNA sequencing data processing, time-series, and DEG analysis.

Bioinformatics analysis was carried out by the Andalusian Bioinformatics Platform of the University of Málaga. Raw data were firstly processed using the in-house customizable pre-processing pipeline SeqTrimNext (96). In this step, clean data (clean reads) were obtained by removing contaminants, sequencing adapters, PolyA/PolyT tails, and short (< 17 nucleotide) and bad quality reads (Phred score < 20); thus, all the downstream analyses were based on this high-quality clean data set. Then, clean reads were mapped to the reference porcine transcriptome (Sus_scrofa. Sscrofa11.1.cdna, with 46.076 unigenes) using Bowtie (v2.2.3) and Samtools (v1.9). The reads count of each transcript was extracted using the python script sam2counts.py (v0.91) (Buffalo, 2010).

Since our data were collected at different time points, a time-series analysis was conducted on clean data using MaSigPro R package (97) to identify clusters of genes with significant temporal expression changes related to 3249 and Lena strain infection over time. Afterwards, DEgenes Hunter R pipeline and DeSeq2 R package (98) were used to identify differentially expressed genes (DEGs) between both infected groups and control group at each time point (1, 3, 6, 8, and 13 dpi). The *P values* were adjusted using Benjamini and Hochberg’s correction for controlling the false discovery rate (FDR). Genes with an FDR < 0.05 and an absolute log_2_ fold change (log2FC) ≥ 1 were assigned as DEGs. The DEGs of Lena- and 3249-infected groups’ data set for each time point were visualized as a volcano plot by using GraphPad Prism (GraphPad Prism software v8.0), underlining the top 5 DEGs with a higher log2FC. Overlapped DEGs at 1, 3, 6, 8, and 13 dpi from Lena- or 3249-infected animals were identified and presented as a Venn diagram using an online tool (http://bioinformatics.psb.ugent.be/webtools/Venn/).

### Gene ontology (GO) and pathway enrichment analysis.

In order to explore GO of DEGs (http://geneontology.org), functional enrichment analyses were conducted using ClueGO (v2.3.3) and CluePedia (v1.3.3), plugins for Cytoscape (v3.8, http://cytoscape.org/) describing biological processes (BPs) and immune system processes (ISPs) GO categories. ClueGO determines the distribution of the DEGs for various GO terms and pathways, generating a functionally grouped GO annotation network (99, 100). The *P* value was calculated using right-sided hypergeometric tests and the Benjamini and Hochberg’s correction for multiple testing (FDR* *<* *0.05). This FDR threshold, together with a high kappa value (0.4), enabled us to precisely select significantly enriched and highly connected GO terms. The most significant term defines the name of the group. In order to avoid over-interpretation of data, a minimum of 3 genes were considered to evaluate the relevance of selected pathways.

### Construction of protein-protein interaction (PPI) network and screening of Hub genes.

The Search Tool for the Retrieval of Interacting Genes (STRING) database was applied to predict PPI and construct a PPI network of selected DEGs (101). Using the STRING database, DEGs with a score ≥ 0.4 were chosen to build a network model visualized by Cytoscape (102). Molecular complex detection (MCODE) is a Cytoscape plugin used to identify the finest PPI sub-network modules (102). The Hub genes are defined as genes with the highest degree of connectivity in the key module. Since the biological networks usually are heterogeneous, it seems to be reasonable to use more than one method for identifying Hub nodes (103); thus, Maximal Clique Centrality (MCC) and Density of Maximum Neighborhood Component (DMNC) algorithms were calculated for each node by CytoHubba plugin in Cytoscape (103). The genes with the top 10 MCC and DMNC values were considered as Hub genes.

### Real-time quantitative reverse transcriptase PCR (RT-qPCR).

To verify the major results drawn from RNA-seq and Hub genes analysis, the expression levels of a panel of 12 identified Hub genes were performed by RT-qPCR. cDNA was synthesized by reverse transcription using iScript cDNA Synthesis Kit (Bio-Rad) from 1 μg of the original RNA sample used for the RNA-seq according to manufacturer’s guidelines. Subsequently, amplifications were run in triplicate using iTaq Universal SYBR Green Supermix (Bio-Rad) on the MyiQ2 Two-Color Real-Time PCR Detection System (Bio-Rad). For each reaction well, 50 ng of cDNA from each animal and 0.5 μM each primer were used. *GAPDH* was chosen as reference gene, and the relative expression level of each Hub gene was calculated by the 2^-ΔΔCt^ method (104). The primers set used for RT-qPCR are listed in Table 5.

**TABLE 5:** Primer sequences of Hub genes used to validate RNA-seq analysis

Gene	Sequences	Reference
*GAPDH*	F 5′-ACATGGCCTCCAAGGAGTAAGA-3″ R 5′-GATCGAGTTGGGGCTGTGACT-3″	105
*CD274* (PD-L1)	F 5′-GTGGAAAAATGTGGCAGCCG-3″ R 5′-TGCTTAGCCCTGACGAACTC-3″	Self-designed
*CTLA4*	F 5′-TCTTCATCCCTGTCTTCTCCAAA-3″ R 5′-GCAGACCCATACTCACACACAAA-3″	106
*HACVR2* (TIM3)	F 5′-TTCGACGGGAGCAGTAAAGC-3″ R 5′-AGGGCAGGACACAGTCAAAG-3″	Self-designed
*LAG3*	F 5-CTCCTCCTGCTCCTTTTGGTT-3″ R 5′-CAGCTCCCCAGTCTTGCTCT-3″	106
*PDCD1* (PD-1)	F 5′- AGCCCAAGCACTTCATCCTC-3″ R 5′- TGTGGAAGTCTCGTCCGTTG-3″	Self-designed
*IL10*	F 5′- TGAGAACAGCTGCATCCACTTC -3″ R 5′- TCTGGTCCTTCGTTTGAAAGAAA -3″	107
*TBX21* (T-bet)	F 5′- TGCAGTCCCTCCATAAGTACCA -3″ R 5′- GCCTCTGGCTCACCATCATT -3″	108
*CD28*	F 5′-CCCCTCAATTCAAGTAACAGGAAAC-3″ R 5′- ATGCCCGGAACTCCTTTGAG-3″	Self-designed
*TNFRSF9*	F 5′-TTGCCAGCAAGGTCAAGAGT-3″ R 5′- AGCCAAAGAACAGTCCGTCC-3″	Self-designed
*GZMB*	F 5′-GCCCCTACATGGCGTATCTTC-3″ R 5′- ACGTTGATTGAGCTTCCCCA-3″	Self-designed
*SELL*	F 5′-CCTAGTCCGATATGTCAAAAACTGG-3′ R 5′- TCATCCATGCTTCTCTGAGACTT-3″	Self-designed
*CCR7*	F 5′-TTGTCATTTTCCAGGTGTGCC-3″ R 5′- GGAGTACATGACCGGGAGGA-3″	Self-designed

### Data availability.

The raw RNA-seq data set was deposited at NCBI Sequence Read Archive database with the accession number PRJNA704925.
